# Discovery of *p*-Terphenyl Metabolites as Potential Phosphodiesterase PDE4D Inhibitors from the Coral-Associated Fungus *Aspergillus* sp. ITBBc1

**DOI:** 10.3390/md20110679

**Published:** 2022-10-28

**Authors:** Zhikai Guo, Ailiman Abulaizi, Ling Huang, Zijun Xiong, Shiqing Zhang, Tianmi Liu, Rong Wang

**Affiliations:** 1 Hainan Key Laboratory of Tropical Microbe Resources, Institute of Tropical Bioscience and Biotechnology, Chinese Academy of Tropical Agricultural Sciences & Key Laboratory for Biology and Genetic Resources of Tropical Crops of Hainan Province, Hainan Institute for Tropical Agricultural Resources, Haikou 571101, China; 2 Hainan Provincial Key Laboratory of Tropical Maricultural Technologies, Hainan Academy of Ocean and Fisheries Sciences, Haikou 571126, China; 3 Hainan Testing Center for the Quality and Safety of Aquatic Products, Hainan Aquatic Technology Extension Station, Haikou 570206, China; 4 Key Laboratory of Tropical Biological Resources of Ministry of Education, School of Pharmaceutical Sciences, Hainan University, Haikou 570208, China; 5 State Key Laboratory of Pharmaceutical Biotechnology, Institute of Functional Biomolecules, School of Life Sciences, Nanjing University, Nanjing 210023, China

**Keywords:** coral-associated fungus, *Aspergillus* sp., natural products, *p*-terphenyls, phosphodiesterase PDE4D inhibitor

## Abstract

Chemical investigation of the fermentation extract of the coral-associated fungus *Aspergillus* sp. ITBBc1 led to the discovery of five unreported *p*-terphenyl derivatives, sanshamycins A–E (**1**–**5**), together with five previously described analogues, terphenyllin (**6**), 3-hydroxyterphenyllin (**7**), candidusin A (**8**), 4,5-dimethoxycandidusin A (**9**), and candidusin C (**10**). Their structures were elucidated by HRESIMS data and NMR spectroscopic analysis. Compound **1** represents the first example of *p*-terphenyls with an aldehyde substitution on the benzene ring. Compounds **2**–**4** feature varying methoxyl and isopentenyl substitutions, while compound **5** features a five-membered lactone linked to a biphenyl. These findings expand the chemical diversity of the family of *p*-terphenyl natural products. Compounds **1**–**6** and **9** were evaluated for their inhibitory activity against type 4 phosphodiesterase (PDE4), which is a fascinating drug target for treatment of inflammatory, respiratory, and neurological diseases. Compound **3** was the most potent and exhibited PDE4D inhibitory activity with an IC_50_ value of 5.543 µM.

## 1. Introduction

Phosphodiesterases (PDEs), the only enzymes that degrade the important secondary messenger 3′,5′-cyclic nucleotides, regulate a myriad of physiological processes in human health and disease [1]. A total of 11 biochemical and pharmacological enzyme families of PDEs (PDE1-PDE11) have been characterized by differences in structure, substrate specificity, inhibitor sensitivities, and tissue distribution [1,2]. Currently they are being explored as important therapeutic targets for treatment of several diseases such as those affecting the respiratory system, nervous system, cardiovascular system, immune system, fertility, and cancer [1]. Type 4 phosphodiesterase (PDE4), a PDE enzyme family that exclusively catalyzes the hydrolysis of the secondary messenger cyclic adenosine 3′,5′-monophosphate (cAMP) in numerous cell types, comprises four subtypes (PDE4A, PDE4B, PDE4C, and PDE4D) with a high degree of sequence identity within the catalytic domains. These enzymes have been reported to be involved in many physiological processes and development of inflammatory, respiratory, autoimmune, neurological diseases, and cancers [1,2,3]. PDE4 has been demonstrated to be a promising drug target for the treatment of chronic obstructive pulmonary disease (COPD), asthma, rheumatoid arthritis, lupus, atopic dermatitis, psoriasis, and neurological disorders [2,3]. Although a number of marketed PDE4 inhibitor drugs have been developed over the last few decades, their side effects, such as nausea, diarrhea, weight loss, and headaches, can not be neglected [2].

**Figure 1:**
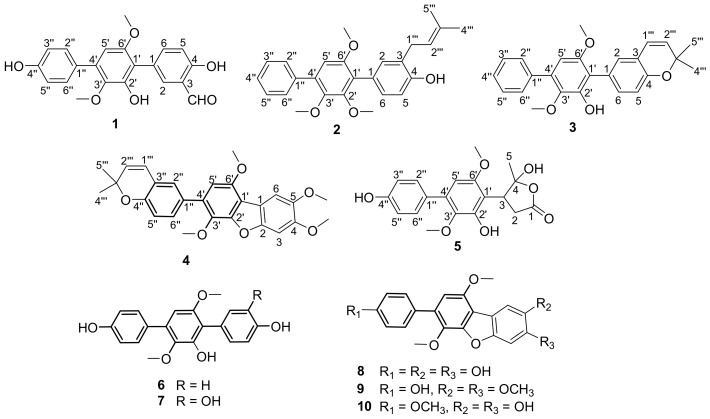
The structures of **1**–**10**.

Since natural products remain a reliable resource for novel drug leads, the search for new natural PDE4 inhibitors is considered to be an attractive project. Recently, naturally occurring PDE4 inhibitors have been reported from several species of plants or marine corals [4,5,6,7]. *p*-Terphenyl natural products are a group of aromatic compounds produced by *Aspergillus* [8,9,10,11,12], *Streptomyces* [13], and *Burkholderia* species [14]. This group of metabolites features a chain of three benzenes with varying substitutions, including hydroxyl, methoxyl and isoprenyl subtituents, and has diverse biological activities including antitumor, antimicrobial, antioxidant, neuraminidase, and phosphodiesterase inhibitory activities [8,9,10,11,12,13,14,15,16]. As part of an ongoing project to identify structurally unique and pharmacologically significant natural products from microbes that are isolated from unexplored or underexplored ecological niches [17,18,19,20,21], our group has obtained a wide variety of structurally diverse and biologically active natural products from marine-derived fungi from the South China Sea [22,23,24,25,26,27,28,29]. During our continuing discovery of novel bioactive secondary metabolites from underexplored fungi from marine resources, five unreported *p*-terphenyl derivatives, namely sanshamycins A–E (**1**–**5**), were isolated along with five previously described analogues (**6**–**10**) from the coral-derived fungus *Aspergillus* sp. ITBBc1 (Figure 1). Structurally, sanshamycin A (**1**) represents the first example of *p*-terphenyls with an aldehyde substitution on the benzene ring and sanshamycins B–D (**2**–**4**) share the same *p*-terphenyl framework with varying methoxyl and isopentenyl substitutions, while sanshamycin E (**5**) features a five-membered lactone linked to a biphenyl. The structures of all the compounds were unambiguously determined by HRESIMS and NMR data. Compounds **1**–**6** and **9** were screened for PDE4D inhibition. Here, we reported the isolation, structure elucidation, and the PDE4D inhibitory activities of these *p*-terphenyls.

## 2. Results

### 2.1. Structure Elucidation of New Compounds ***1***–***5***

**Figure 2:**
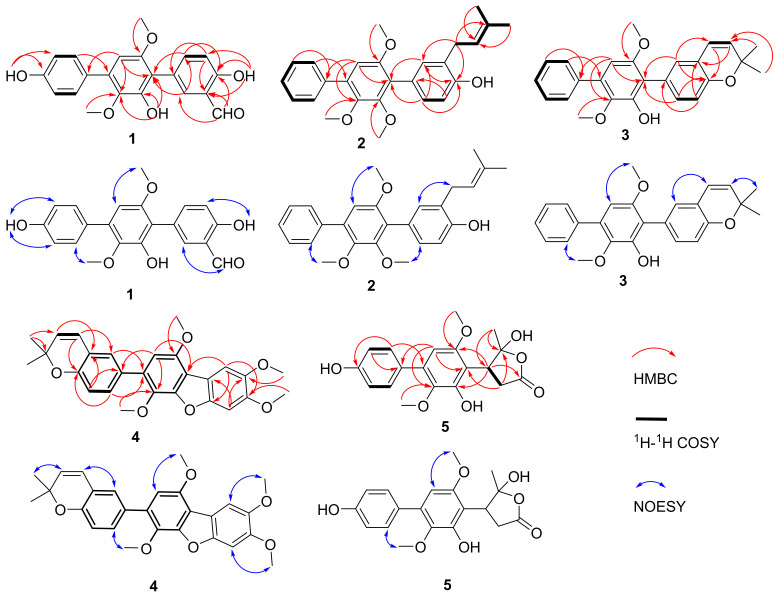
Key HMBC, ^1^H-^1^H COSY and ROESY correlations of **1**–**5**.

Sanshamycin A (**1**) was isolated as a yellow powder. The molecular formula was established as C_21_H_18_O_6_ (13 degrees of unsaturations) on the basis of its HRESIMS (Figure S8) and the ^1^H and ^13^C NMR data (Table 1 and Table 2). The initial analysis of the ^1^H (Figure S1), ^13^C (Figure S2), DEPT135 (Figure S3), and HSQC (Figure S4) spectra in acetone-*d*_6_ revealed that **1** was very similar to terphenyllin (**6**) [8], but with an aldehyde group (3-CHO) displaying characteristic proton and carbon signals at *δ*_H_ 10.06 (s) and *δ*_C_ 197.3. Further observation of the key HMBC correlations (Figure 2 and Figure S5) from *δ*_H_ 10.06 proton signal to C-2 (*δ*_C_ 136.2), C-3 (*δ*_C_ 120.6) and C-4 (*δ*_C_ 160.1), from H-2 (*δ*_H_ 7.83, d, *J* = 2.2 Hz) to *δ*_C_ 197.3 carbon signal, and the NOESY correlation (Figure 2 and Figure S7) from *δ*_H_ 10.06 proton signal to H-2 located the aldehyde group at C-3. Characteristic HMBC correlations from 4-OH (*δ*_H_ 11.02, s) to C-3, C-4, and C-5 (*δ*_C_ 116.1), 2′-OH (*δ*_H_ 7.90, s) to C-1′ (*δ*_C_ 114.8), C-2′ (*δ*_C_ 148.3), and C-3′ (*δ*_C_ 139.3), 4′′-OH (*δ*_H_ 8.55, s) to C-3′′ (*δ*_C_ 115.2), C-4′′ (*δ*_C_ 157.1), and C-5′′ (*δ*_C_ 115.2), 3′-OCH_3_ (*δ*_H_ 3.40, s) to C-3′, 6′-OCH_3_ (*δ*_H_ 3.78, s) to C-6′ (*δ*_C_ 153.5) were observed. Meanwhile, the ROESY spectrum showed correlations from 4-OH to H-5 (*δ*_H_ 7.02, d, *J* = 8.6 Hz), 3′-OCH_3_ to 2′-OH, H-2′′ (*δ*_H_ 7.55, d, *J* = 8.6 Hz) and H-6′′ (*δ*_H_ 7.55, d, *J* = 8.6 Hz), and 4′′-OH to H-3′′ (*δ*_H_ 6.96, d, *J* = 8.6 Hz) and H-5′′ (*δ*_H_ 6.96, d, *J* = 8.6 Hz). Further comprehensive interpretation of the HSQC, HMBC, ^1^H-^1^H COSY (Figure S6), and ROESY data allowed for the full assignment of the structure as shown in Figure 1.

Sanshamycin B (**2**) was isolated as a yellowish powder, and had the molecular formula C_26_H_28_O_4_ on the basis of the HRESIMS (Figure S18) and 1D NMR data (Table 1 and Table 2). Analysis of the ^1^H (Figure S11), ^13^C (Figure S12), DEPT135 (Figure S13) and HSQC (Figure S14) NMR data of **2** showed the presence of three methoxyl groups, two methyl groups, one methylene, and 20 sp^2^ carbons (ten of which were protonated). Analysis of the ^13^C NMR data, the coupling constants of the aromatic protons in the ^1^H NMR spectrum and also the correlations observed in the COSY spectrum revealed the presence of a pentasubstituted, a 1,3,4-trisubstituted, and a monosubstituted benzene rings, indicating a *p*-terphenyl framework structure similar to **1**. The presence of an isopentyl group could be deduced from HMBC correlations (Figure S15) from H_2_-1′′′ (*δ*_H_ 3.40, d, *J* = 7.2 Hz), H_3_-4′′′ (*δ*_H_ 1.78, d, *J* = 1.4 Hz) and H_3_-5′′′ (*δ*_H_ 1.79, br s) to C-3′′′ (*δ*_C_ 134.7), H_3_-4′′′ and H_3_-5′′′ to C-2′′′ (*δ*_C_ 122.0), ^1^H-^1^H COSY correlations (Figure S16) from H_2_-1′′′ to H-2′′′ (*δ*_H_ 5.40, dq, *J* = 7.2, 1.4 Hz), and NOESY correlations (Figure S17) from H_2_-1′′′ to H_3_-5′′′ and H-2′′′ to H_3_-4′′′. That the isopentyl moiety was located at C-3 was evidenced from HMBC correlations from H_2_-1′′′ to C-2 (*δ*_C_ 132.1), C-3 (*δ*_C_ 125.9), and C-4 (*δ*_C_ 153.5), and NOESY correlation from H_2_-1′′′ to H-2 (*δ*_H_ 7.17, d, *J* = 2.1 Hz). Characteristic HMBC correlations from the methoxyl group at *δ*_H_ 3.66 to C-2′ (*δ*_C_ 152.0), the methoxyl group at *δ*_H_ 3.61 to C-3′ (*δ*_C_ 144.8) and the methoxyl group at *δ*_H_ 3.74 to C-6′ (*δ*_C_ 153.2) and NOESY correlations between 2′-OCH_3_ and H-6 (*δ*_H_ 7.17, overlap), between 3′-OCH_3_ and H-2′′(H-6′′) (*δ*_H_ 7.59, d, *J* = 7.5 Hz), and between 6′-OCH_3_ and H-5′ (*δ*_H_ 6.69, s), confirmed the positions of these methoxyl groups. The key HSQC, HMBC, ^1^H-^1^H COSY and ROESY of **2** was shown in Figure 2.

Sanshamycin C (**3**), obtained as a yellow powder, gave a molecular formula of C_25_H_24_O_4_, as determined by HRESIMS (Figure S28), and its ^1^H and ^13^C NMR data (Table 1 and Table 2), resembled those for sanshamycin B (**2**). The major differences observed in the ^1^H NMR spectrum (Figure S21) for **3** relative to those of **2** indicated the presence of an cis-disubstituted double bond due to the coupling constants of protons at *δ*_H_ 6.35 (d, *J* = 9.7 Hz, H-1′′′) and 5.60 (d, *J* = 9.7 Hz, H-2′′′) instead of a methylene signal at *δ*_H_ 3.40 and an olefinic methine signal at *δ*_H_ 5.40, and the absence of a methoxyl subtituent in **3**. Comprehensive consideration of the HRESIMS and NMR data suggested **3** could be derived from the cyclization between the hydroxyl group 4-OH and C-3′′′ of the isopentyl group of **2**. This suggestion was confirmed by HMBC correlations (Figure S25) from H-1′′′ to C-4 (*δ*_C_ 152.3) and C-3′′′ (*δ*_C_ 76.4), H-2′′′ to C-3 (*δ*_C_ 120.8), H-4′′′ (*δ*_H_ 1.47, s) and H-5′′′ (*δ*_H_ 1.47, s) to C-2′′′ (*δ*_C_ 130.3) and C-3′′′, H-2 (*δ*_H_ 7.09, d, *J* = 2.1 Hz) to C-1′′′ (*δ*_C_ 122.4), and NOESY correlation (Figure S27) from H-1′′′ to H-2. Correlations from the methoxyl group at *δ*_H_ 3.44 to C-3′ (*δ*_C_ 138.9) and the methoxyl group at *δ*_H_ 3.75 to C-6′ (*δ*_C_ 153.5) in the HMBC spectrum and NOESY correlations from 3′-OCH_3_ to H-2′′/H-6′′ (*δ*_H_ 7.64), and from 6′-OCH_3_ to H-5′ (*δ*_H_ 6.48, s) were observed. Overall analysis of the 1D and 2D NMR data permitted the structural assignment for **3** as showing in Figure 1.

Sanshamycin D (**4**) was isolated as a white powder whose molecular formula of C_27_H_26_O_6_ was obtained by analysis of HRESIMS (Figure S38) and NMR data (Figures S31–S37). The ^1^H and ^13^C NMR data of **4** were very similar to those of 4,5-dimethoxycandidusin A (**9**) [11], except for the presence of a prenyl and the absence of OH-4′′. The prenyl moiety was elucidated by HMBC correlations from H-4′′′/H-5′′′ (*δ*_H_ 1.49, s) to C-2′′′ (*δ*_C_ 130.8) and C-3′′′ (*δ*_C_ 76.5), and ^1^H-^1^H COSY correlations from H-1′′′ (*δ*_H_ 6.40, d, *J* = 9.7 Hz) to H-2′′′ (*δ*_H_ 5.66, d, *J* = 9.7 Hz). That the prenyl group was at C-3′′ was evidenced from the HMBC correlations from H-1′′′ to C-2′′ (*δ*_C_ 127.4) and C-4′′ (*δ*_C_ 152.3), H-2′′′ to C-3′′ (*δ*_C_ 121.0), and NOESY correlation from H-1′′′ to H-2′′ (*δ*_H_ 7.25, s). The HMBC spectrum displayed correlations from the methoxyl signals at *δ*_H_ 3.98, 4.02, 3.71, and 4.03 to C-4, C-5, C-3′, and C-6′, respectively. Significant NOESY correlations from 4-OCH_3_ to H-3 (*δ*_H_ 7.17, s), 5-OCH_3_ to H-6 (*δ*_H_ 7.56, s), 3′-OCH_3_ to H-6′′ (*δ*_H_ 7.38, d, *J* = 8.3 Hz), and 6′-OCH_3_ to H-5′ (*δ*_H_ 6.68, s), secured the positions of these methoxyl groups. Complete NMR analysis supported the full elucidation of the structure for compound **4** as shown in Figure 2. 

Sanshamycin E (**5**) was isolated as a white powder. Its molecular formula was determined as C_19_H_20_O_7_ (10 degrees of unsaturations) by HRESIMS (Figure S48) in combination with ^1^H and ^13^C NMR data. The ^1^H (Figure S41), ^13^C (Figure S42), DEPT135 (Figure S43), and HSQC (Figure S44) NMR spectra (Table 1 and Table 2) showed resonances for 19 carbons, including 12 sp^2^ carbons (five of which are protonated), one ester carbonyl carbon, one quaternary sp^3^ carbon, two methoxyl carbons, one methylene sp^2^ carbon, one methine sp^3^ carbon, and one methyl carbon. Detailed analysis of the coupling constants of the protons in the ^1^H NMR spectrum and COSY correlations indicated the presence of a pentasubstituted and a 1,4-disubstituted benzene rings. Characteristic HMBC correlations (Figure S45) from H-2′′/H-6′′ (*δ*_H_ 7.41, d, *J* = 8.5 Hz) to C-4′′ (*δ*_C_ 155.1) and C-4′ (*δ*_C_ 136.4) and from H-3′′/H-5′′ (*δ*_H_ 6.90, d, *J* = 8.5 Hz) and H-5′ (*δ*_H_ 6.43, s) to C-1′′ (*δ*_C_ 130.5) secured the connectivity of C-1′′ to C-4′. HMBC correlations from the methoxyl proton signals at *δ*_H_ 3.66 to C-3′ (*δ*_C_ 136.0), at *δ*_H_ 3.84 to C-6′ (*δ*_C_ 151.5), from H-5′ to C-3′ and C-6′, and NOESY correlations (Figure S47) from 6′-OCH_3_ to H-5′ and from 3′-OCH_3_ to H-2′′/H-6′′ confirmed the locations of the methoxyl groups at C-3′ and C-6′. So far, nine degrees of unsaturation have been assigned, while the last degree of unsaturation could be completed by the presence of a γ-butyrolactone moiety, which was deduced from the HMBC correlations from H_2_-2 (*δ*_H_ 3.08, dd, *J* = 6.4, 4.5 Hz) to C-1 (*δ*_C_ 173.9) and C-4 (*δ*_C_ 118.3), H-3 (*δ*_H_ 3.96, dd, *J* = 6.4, 4.5 Hz) to C-1 and C-5 (*δ*_C_ 23.9), H_3_-5 (*δ*_H_ 1.94, s) to C-3 (*δ*_C_ 45.7) and C-4, and ^1^H-^1^H COSY correlation (Figure S46) from H_2_-2 to H-3. The high chemical shift value (*δ*_C_ 118.3) of C-4 is a characteristic of ketal carbon, which could suggest the connectivity of C-1 and C-4 through a ester bond. The γ-butyrolactone moiety was connected with the biphenyl moiety, which could be deduced from the HMBC correlations from H_2_-2 to C-1′ (*δ*_C_ 114.6) and from H-3 to C-2′ (*δ*_C_ 149.9) and C-6′. We have tried to determine the absolute configurations of C-3 and C-4 by comparing the experimental and calculated ECD data, but unable to reach a conclusion. Finally, the structure for **5** was determined as shown in Figure 1. A plausible biosynthetic pathway for **5** was tentatively proposed in the Supplementary Materials (Figure S51).

The known compounds terphenyllin (**6**) [8], 3-hydroxyterphenyllin (**7**) [9], candidusin A (**8**) [10], 4,5-dimethoxycandidusin A (**9**) [11], and candidusin C (**10**) [12] were identified by comparison of their NMR data with those reported in the literature.

### 2.2. In Vitro Evaluation of Type 4 Phosphodiesterase PDE4D Inhibitory Activity

*p*-Terphenyl compounds have been identified as phosphodiesterase (PDE) inhibitors. Terferol, derived from *Streptomyces showdoensis* SANK 65080, showed inhibitory activity towards cyclic adenosine 3′,5′-monophosphate phosphodiesterase (cAMP-PDE) and cyclic guanosine 3′,5′-monophosphate phosphodiesterase (cGMP-PDE) from various rat tissues [15]. Moreover, one metabolite with a similar structure to terferol from two different microbes was also found to possess inhibitory activities towards eukaryotic PDE11 and four PDE4s [14,16]. Given the close structural relationship of **1**–**10** with the known natural phosphodiesterase inhibitor terferol, the inhibitory activities of **1**–**6** and **9** were evaluated against the PDE4D, with rolipram as the positive control. As shown in Table 3, **3** was the most potent compound and displayed the best inhibition at 5 µM with inhibitory percentage of 49.4 %, while the other compounds showed weaker activities with inhibitory percentage of 4.8–23.2% (Table 3). Compound **3** was selected to test the concentration required for 50% inhibition of PDE4D (IC_50_), which gave an IC_50_ value of 5.543 ± 0.24 µM, while the positive drug rolipram exhibited an IC_50_ value of 0.588 ± 0.057 µM. Comparing with the other tested compounds, the two fused six-membered rings and the hydroxyl group at C-2′ in **3** may be responsible for the strong PDE4D inhibitory activity.

## 3. Materials and Methods

### 3.1. General Experimental Procedures

One- and two-dimensional NMR spectra were measured on Bruker AVIII-500 NMR spectrometer (Bruker Corporation, Karlsruhe, Germany). The chemical shifts of ^1^H (500 MHz) and ^13^C (125 MHz) NMR data were given in *δ* (ppm) and referenced to the solvent signal (CDCl_3_, *δ*_H_ 7.26 and *δ*_C_ 77.16; acetone-*d*_6_, *δ*_H_ 2.05 and *δ*_C_ 29.84). HRESIMS data were collected on an Agilent 6210 TOF LC-MS instrument (Agilent Technologies Inc., Palo Alto, CA, USA). Optical rotation value was recorded by a JASCO P-1020 digital polarimeter (JASCO, Tokyo, Japan). UV and IR data were measured on a UV-2550 spectrometer (Shimadzu, Kyoto, Japan) and Nicolet 380 Infrared Spectrometer (Thermo Electron Corporation, Madison, WI, USA), respectively. The ECD data were collected on JASCO J-715 spectropolarimeter (JASCO, Tokyo, Japan). The semi-preparative HPLC was conducted on a Waters 1525 HPLC equipped with a XBridge C18 column (5 μm, 250.0 mm × 10.0 mm; Waters Corporation, Milford, MA, USA). Thin-layer chromatography (TLC) was performed on pre-coated glass plates (silica gel GF_254_, Qingdao Marine Chemical Inc., Qingdao, China). Column chromatography (CC) was performed on silica gel (45–75 µm; Qingdao Marine Chemical Inc., Qingdao, China), ODS (40–60 µm; Osaka Soda Co., Ltd., Hyogo, Japan) and Sephadex LH-20 (Cytiva, Uppsala, Sweden).

### 3.2. Fungal Material and Fermentation

The fungal strain ITBBc1 was isolated from a coral reef in the South China Sea in Sansha, Hainan, China. This fungal strain was identified as *Aspergillus* sp. by internal transcribed spacer (ITS) sequence (GenBank accession number OP614945). The voucher specimen of this strain was deposited in the Hainan Key Laboratory of Tropical Microbe Resources, Institute of Tropical Bioscience and Biotechnology, Haikou, P.R. China. The strain was cultivated on a PDA agar plate (consisting of potato extract 200 g/L, glucose 20 g/L, agar 15 g/L, chloramphenicol 0.1 g/L and 1 L sterilized deionized water) at 28 °C for 5 days. Then, the agar plugs with mycelia were added into 1 L Erlenmeyer flasks, each containing 200 mL ME liquid medium (consisting of malt extract 10.0 g/L, sucrose 10.0 g/L, peptone 1.0 g/L and 1 L sterilized deionized water), which was cultivated on a rotary shaker at 160 rpm/min at 28 °C. After 4 days of fermentation, 15 mL of the seed cultures were inoculated into the rice solid media (consisting of rice 30 g and seawater 45 mL) in 1L-Erlenmeyer flasks and fermented at 28 °C for 45 days under static conditions.

### 3.3. Extraction and Isolation

The whole fermentation materials of strain ITBBc1 were collected and extracted with ethyl acetate (EtOAc) at room temperature for four times to yield a crude extract (120.0 g). Then, the crude extract was fractionated by silica gel CC using gradient elution of petroleum ether/EtOAc mixtures (*v/v*, 100:1, 50:1, 25:1, 10:1, 5:1, 2:1, 1:1, 0:1) to give 8 fractions (Fr.A–Fr.H). The fraction Fr.E (petroleum ether/EtOAc, *v/v*, 5:1) was separated by ODS reversed-phase CC eluted with aqueous methanol (40% to 100%) to yield the Fr.E7 (85% aqueous methanol), which was further purified by Sephadex LH-20 (eluted with 100% methanol) and then silica gel CC [using gradient elution of petroleum ether/EtOAc mixtures (*v/v*, 53:1, 25:1, 10:2)] to give compounds **2** (6.6 mg), **3** (5.0 mg) and **4** (2.2 mg). The fraction Fr.F (petroleum ether/EtOAc, *v/v*, 2:1) was subjected to ODS reversed-phase CC eluted with aqueous methanol (45% to 100%) to produce nine subfractions Fr.F1–F9. The Fr.F1 (45% aqueous methanol) was further subjected to Sephadex LH-20 (eluted with 100% methanol) and then silica gel CC [using gradient elution of petroleum ether/EtOAc mixtures (*v/v*, 5:3, 1:1)] to afford compound **5** (2.5 mg). The Fr.F3 (55% aqueous methanol) was purified by Sephadex LH-20 (eluted with 100% methanol) to give compound **1** (3.5 mg). The Fr.F5 (65% aqueous methanol) was subjected to Sephadex LH-20 (eluted with 100% methanol) and then purified by semi-preparative reverse-phase HPLC (eluted with 70% aqueous methanol; 3 mL/min, UV *λ*_max_ 254 nm) to give compound **10** (42.1 mg, *t*_R_ 9.1 min). The Fr.F6 (75% aqueous methanol) was purified by Sephadex LH-20 (eluted with 100% methanol) to give compound **9** (0.9 mg). Compound **6** was recrystallized from the fraction Fr.G (petroleum ether/EtOAc, *v/v*, 1:1) and obtained as white crystals (90 mg). Then, the rest of the Fr.G was subjected to ODS reversed-phase CC eluted with aqueous methanol (40% to 100%) to yield seven subfractions Fr.G1–G7. The Fr.G3 (50% aqueous methanol) was separated by Sephadex LH-20 (eluted with 100% methanol) and then purified by semi-preparative reverse-phase HPLC (3 mL/min, UV *λ*_max_ 254 nm) to give compounds **7** (59.1 mg, *t*_R_ 5.5 min; eluted with 60% aqueous methanol) and **8** (13.2 mg, *t*_R_ 8.5 min; eluted with 65% aqueous methanol).

Sanshamycin A (**1**): yellow powder; UV (MeOH) *λ*_max_ (log *ε*): 304 (2.81), 332 (2.52), 342 (2.54) nm; IR(KBr) *ν*_max_: 3415, 2918, 1652, 1403, 1118, 1075, 835, 759 cm^−1^; ^1^H and ^13^C NMR data, see Table 1 and Table 2; HRESIMS *m/z* 389.1001 [M + Na]^+^ (calculated for C_21_H_18_NaO_6_, 389.0996).
Sanshamycin B (**2**): yellowish powder; UV (MeOH) *λ*_max_ (log *ε*): 258 (2.60), 298 (2.81) nm; IR(KBr) *ν*_max_: 3404, 2931, 1599, 1465, 1389, 1111, 1079, 1025, 616, 547 cm^−1^; ^1^H and ^13^C NMR data, see Table 1 and Table 2; HRESIMS *m/z* 427.1928 [M + Na]^+^ (calculated for C_26_H_28_NaO_4_, 427.1880).
Sanshamycin C (**3**): yellow powder; UV (MeOH) *λ*_max_ (log *ε*): 273 (2.68), 302 (2.83) nm; IR(KBr) *ν*_max_: 3416, 2934, 2434, 1629, 1468, 1404, 1123, 1072, 795 cm^−1^; ^1^H and ^13^C NMR data, see Table 1 and Table 2; HRESIMS *m/z* 389.1752 [M + H]^+^ (calculated for C_25_H_25_O_4_, 389.1747).
Sanshamycin D (**4**): white powder; UV (MeOH) *λ*_max_ (log *ε*): 258 (2.64), 310 (2.92), 337 (2.96) nm; IR(KBr) *ν*_max_: 3411, 2919, 1617, 1480, 1384, 1129, 1023, 617 cm^−1^; ^1^H and ^13^C NMR data, see Table 1 and Table 2; HRESIMS *m/z* 447.1801 [M + H]^+^ (calculated for C_27_H_27_O_6_, 447.1802).
Sanshamycin E (**5**): white powder; ${\left[\alpha \right]}_{D}^{25}$ 68 (*c* 0.10, MeOH); UV (MeOH) *λ*_max_ (log *ε*): 231 (2.50), 266 (2.67) nm; IR(KBr) *ν*_max_: 3430, 2922, 1628, 1382, 1097, 638, 534 cm^−1^; ECD (MeOH) *λ*_max_ (∆ε): 190 (−19.29), 199 (+21.27), 208 (+17.37), 234 (−0.33) nm; ^1^H and ^13^C NMR data, see Table 1 and Table 2; HRESIMS *m/z* 383.1080 [M + Na]^+^ (calculated for C_19_H_20_NaO_7_, 383.1101).

### 3.4. Type 4 Phosphodiesterase PDE4D Inhibitiory Screening Assay

The type 4 phosphodiesterase PDE4D inhibitor assays were performed as described previously [30,31,32]. The inhibition of PDE4D by compounds **1**–**6** and **9** were assayed by a PerkinElmer 2910 (PerkinElmer, Inc., Waltham, MA, USA) liquid scintillation counter. Rolipram was used as a positive drug. Three independent experiments were conducted for the measurement of the inhibitory effect of compound **3** against PDE4D. The experimental data were analyzed via GraphPad Prism 5.1 (GraphPad Software, San Diego, CA, USA), and the IC_50_ values were calculated by nonlinear regression. 

## 4. Conclusions

In conclusion, five new *p*-terphenyl derivatives, sanshamycins A–E (**1**–**5**), and five known analogues (**6**–**10**) were isolated and characterized from the coral-associated fungus *Aspergillus* sp. ITBBc1. The structures of the unreported compounds (**1**–**5**) were elucidated by interpretation of their 1D and 2D NMR and HRESIMS data whiles the structures of the previously reported compounds (**6**–**10**) were identified by comparison of their NMR data with those reported in literature. Sanshamycin A (**1**) represents the first example of *p*-terphenyls with an aldehyde substitution on the benzene ring. Sanshamycins B–D (**2**–**4**) feature varying methoxyl and isopentenyl substitutions, while sanshamycin E (**5**) features a five-membered lactone linked to a biphenyl. The inhibitory effects of **1**–**6** and **9** on PDE4D were assessed in vitro and **3** exhibited potent PDE4D inhibitory activity with an IC_50_ value of 5.543 µM. The current study revealed a new natural type 4 phosphodiesterase PDE4 inhibitor from the marine-derived fungus, *Aspergillus* sp. strain ITBBc1, could serve as a new structural motif for the future PDE4 inhibitor design. In general, the results of this study expand the knowledge of the chemical and biological diversity of the family of *p*-terphenyl natural products, and may provide a promising lead structure for the future development of PDE4 inhibitors. The inhibitory activity against other PDE members and the PDE4-inhibitory mechanism of sanshamycin C (**3**) deserves further investigation.

## Figures and Tables

**Table 1:** ^1^H NMR (500 MHz) spectroscopic data for **1**–**5**.

Position	1 ^a^	2 ^b^	3 ^b^	4 ^b^	5 ^b^
2	7.83, d (2.2)	7.17, d (2.1)	7.09, d (2.1)		3.08, dd (6.4, 4.5)
3				7.17, s	3.96, dd (6.4, 4.5)
3-CHO	10.06, s				
4-OH	11.02, s				
4-OCH_3_				3.98, s	
5	7.02, d (8.6)	6.87, d (8.7)	6.85, d (8.2)		1.94, s
5-OCH_3_				4.02, s	
6	7.68, dd (8.6, 2.2)	7.17, overlapping	7.22, dd (8.2, 2.1)	7.56, s	
2′-OH	7.90, s		5.92, (br s)		
2′-OCH_3_		3.66, s			
3′-OCH_3_	3.40, s	3.61, s	3.44, s	3.71, s	3.66, s
5′	6.55, s	6.69, s	6.48, s	6.68, s	6.43, s
6′-OCH_3_	3.78, s	3.74, s	3.75, s	4.03, s	3.84, s
2′′	7.55, d (8.6)	7.59, d (7.5)	7.64, m	7.25, s	7.41, d (8.5)
3′′	6.96, d (8.6)	7.45, t (7.5)	7.46, t (7.5)		6.90, d (8.5)
4′′		7.37, t (7.5)	7.37, t (7.5)		
4′′-OH	8.55, s				
5′′	6.96, d (8.6)	7.45, t (7.5)	7.46, t (7.5)	6.86, d (8.3)	6.90, d (8.5)
6′′	7.55, d (8.6)	7.59, d (7.5)	7.64, m	7.38, d (8.3)	7.41, d (8.5)
1′′′		3.40, d (7.2)	6.35, d (9.7)	6.40, d (9.7)	
2′′′		5.40, dq (7.2, 1.4)	5.60, d (9.7)	5.66, d (9.7)	
4′′′		1.78, d (1.4)	1.47, s	1.49, s	
5′′′		1.79, br s	1.47, s	1.49, s	

^a^ Spectra were recorded in acetone-*d*_6_; ^b^ Spectra were recorded in CDCl_3_. *δ* in ppm.

**Table 2:** ^13^C NMR (125 MHz) spectroscopic data for **1**–**5**.

Position	1 ^a^	2 ^b^	3 ^b^	4 ^b^	5 ^b^
1	126.1, C	126.1, C	125.0, C	115.3, C	173.9, C
2	136.2, CH	132.1, CH	128.7, CH	150.6, C	34.0, CH_2_
3	120.6, C	125.9, C	120.8, C	95.4, CH	45.7, CH
3-CHO	197.3, CH				
4	160.1, C	153.5, C	152.3, C	149.1, C	118.3, C
4-OCH_3_				56.3, CH_3_	
5	116.1, CH	115.2, CH	116.0, CH	146.2, C	23.9, CH_3_
5-OCH_3_				56.6, CH_3_	
6	140.0, CH	130.0, CH	131.5, CH	104.3, CH	
1′	114.8, C	124.7, C	116.7, C	114.8, C	114.6, C
2′	148.3, C	152.0, C	147.2, C	149.3, C	149.9, C
2′-OCH_3_		60.8, CH_3_			
3′	139.3, C	144.8, C	138.9, C	136.7, C	136.0, C
3′-OCH_3_	59.8, CH_3_	60.9, CH_3_	60.9, CH_3_	61.1, CH_3_	60.7, CH_3_
4′	133.4, C	134.7, C	132.7, C	131.1, C	136.4, C
5′	103.3, CH	108.1, CH	104.0, CH	105.6, CH	106.1, CH
6′	153.5, C	153.2, C	153.5, C	150.0, C	151.5, C
6′-OCH_3_	55.3, CH_3_	56.1, CH_3_	56.0, CH_3_	55.9, CH_3_	56.0, CH_3_
1′′	129.4, C	138.5, C	138.1, C	131.0, C	130.5, C
2′′	129.9, CH	129.2, CH	128.8, CH	127.4, CH	130.5, CH
3′′	115.2, CH	128.2, CH	128.5, CH	121.0, C	115.1, CH
4′′	157.1, C	127.3, CH	127.5, CH	152.3, C	155.1, C
5′′	115.2, CH	128.2, CH	128.5, CH	116.1, CH	115.1, CH
6′′	129.9, CH	129.2, CH	128.8, CH	130.2, CH	130.5, CH
1′′′		29.9, CH_2_	122.4, CH	122.4, CH	
2′′′		122.0, CH	130.3, CH	130.8, CH	
3′′′		134.7, C	76.4, C	76.5, C	
4′′′		25.8, CH_3_	28.4, CH_3_	28.2, CH_3_	
5′′′		17.9, CH_3_	28.4, CH_3_	28.2, CH_3_	

^a^ Spectra were recorded in acetone-*d*_6_; ^b^ Spectra were recorded in CDCl_3_. *δ* in ppm.

**Table 3:** Inhibitory activities of **1**–**6** and **9** towards PDE4D.

Compounds	PDE4D Inhibitory Percentage (100%)
Rolipram	52.0
**1**	4.8
**2**	6.7
**3**	49.4
**4**	12.4
**5**	5.1
**6**	12.8
**9**	23.2

The test concentration of positive rolipram is 0.5 μM; The test concentrations of **1**–**6** and **9** are 5 μM.

## Data Availability

The authors declare that all relevant data supporting the results of this study are available within the article and its Supplementary Materials file, or from the corresponding authors upon request.

## Supplementary Materials

The following supporting information can be downloaded at: https://www.mdpi.com/article/10.3390/md20110679/s1, Figures S1–S51: 1D, 2D NMR, MS, UV, and IR spectra of compounds **1**–**5**.
