# Supplementary material for: Discovery of p-Terphenyl Metabolites as Potential Phosphodiesterase PDE4D Inhibitors from the Coral-Associated Fungus Aspergillus sp. ITBBc1
Source: Mar Drugs. 2022 Oct 28;20(11):679. doi: 10.3390/md20110679 (PMC9696254; doi:10.3390/md20110679)

Supporting Information

Discovery of *p*-Terphenyl Metabolites as Potential Phosphodiesterase PDE4D Inhibitors from the Coral-Associated Fungus *Aspergillus* sp. ITBBc1

Zhikai Guo [1,*], Ailiman Abulaizi [5], Ling Huang [4], Zijun Xiong [1], Shiqing Zhang [1], Tianmi Liu [3,*], Rong Wang [2,*]

[1] Hainan Key Laboratory of Tropical Microbe Resources, Institute of Tropical Bioscience and Biotechnology, Chinese Academy of Tropical Agricultural Sciences & Key Laboratory for Biology and Genetic Resources of Tropical Crops of Hainan Province, Hainan Institute for Tropical Agricultural Resources, Haikou 571101, China

[2] Hainan Provincial Key Laboratory of Tropical Maricultural Technologies, Hainan Academy of Ocean and Fisheries Sciences, Haikou 571126, China

[3] Hainan Testing Center for the Quality and Safety of Aquatic Products, Hainan Aquatic Technology Extension Station, Haikou 570206, China

[4] Key Laboratory of Tropical Biological Resources of Ministry of Education, School of Pharmaceutical Sciences, Hainan University, Haikou 570208, China

[5] State Key Laboratory of Pharmaceutical Biotechnology, Institute of Functional Biomolecules, School of Life Sciences, Nanjing University, Nanjing 210023, China

* Correspondence: guozhikai@itbb.org.cn (Z.G.); ltm600@163.com (T.L.); wangrong1982@gmail.com (R.W.)

Figure S1. ^{1}H NMR (500 MHz, acetone-d_6) spectrum of compound **1**.

Figure S2. ^{13}C NMR (125 MHz, acetone-d_6) spectrum of compound **1.**

Figure S3. DEPT135 spectrum of compound **1.**

Figure S4. HSQC spectrum of compound **1.**

Figure S5. HMBC spectrum of compound **1.**

Figure S6. ^{1}H-^{1}H COSY spectrum of compound **1.**

Figure S7. ROESY spectrum of compound **1.**

Figure S8. The HRESIMS spectrum of compound **1.**

Figure S9. UV spectrum of compound **1.**

Figure S10. IR spectrum of compound **1.**

Figure S11. ^{1}H NMR (500 MHz, $CDCl_3$) spectrum of compound **2**.

Figure S12. ^{13}C NMR (125 MHz, $CDCl_3$) spectrum of compound **2**.

Figure S13. DEPT135 spectrum of compound **2.**

Figure S14. HSQC spectrum of compound **2.**

Figure S15. HMBC spectrum of compound **2.**

Figure S16. ^{1}H-^{1}H COSY spectrum of compound **2.**

Figure S17. ROESY spectrum of compound **2.**

Figure S18. The HRESIMS spectrum of compound **2.**

Figure S19. UV spectrum of compound **2.**

Figure S20. IR spectrum of compound **2.**

Figure S21. ^{1}H NMR (500 MHz, $CDCl_3$) spectrum of compound **3**.

Figure S22. ^{13}C NMR (125 MHz, $CDCl_3$) spectrum of compound **3.**

Figure S23. DEPT135 spectrum of compound **3.**

Figure S24. HSQC spectrum of compound **3.**

Figure S25. HMBC spectrum of compound **3.**

Figure S26. ^{1}H-^{1}H COSY spectrum of compound **3.**

Figure S27. ROESY spectrum of compound **3.**

Figure S28. The HRESIMS spectrum of compound **3.**

Figure S29. UV spectrum of compound **3.**

Figure S30. IR spectrum of compound **3.**

Figure S31. ^{1}H NMR (500 MHz, $CDCl_3$) spectrum of compound **4**.

Figure S32. ^{13}C NMR (125 MHz, $CDCl_3$) spectrum of compound **4**.

Figure S33. DEPT135 spectrum of compound **4.**

Figure S34. HSQC spectrum of compound **4.**

Figure S35. HMBC spectrum of compound **4.**

Figure S36. ^{1}H-^{1}H COSY spectrum of compound **4.**

Figure S37. ROESY spectrum of compound **4.**

Figure S38. The HRESIMS spectrum of compound **4.**

Figure S39. UV spectrum of compound **4.**

Figure S40. IR spectrum of compound **4.**

Figure S41. ^{1}H NMR (500 MHz, $CDCl_3$) spectrum of compound **5**.

Figure S42. ^{13}C NMR (125 MHz, $CDCl_3$) spectrum of compound **5.**

Figure S43. DEPT135 spectrum of compound **5.**

Figure S44. HSQC spectrum of compound **5.**

Figure S45. HMBC spectrum of compound **5.**

Figure S46. ^{1}H-^{1}H COSY spectrum of compound **5.**

Figure S47. NOESY spectrum of compound **5.**

Figure S48. The HRESIMS spectrum of compound **5.**

Figure S49. UV spectrum of compound **5.**

Figure S50. IR spectrum of compound **5.**

Figure S51. Predicted biosynthetic pathway for compound **5.**

Figure S1 ^{1}H NMR (500 MHz, acetone-d_6) spectrum of compound **1**.

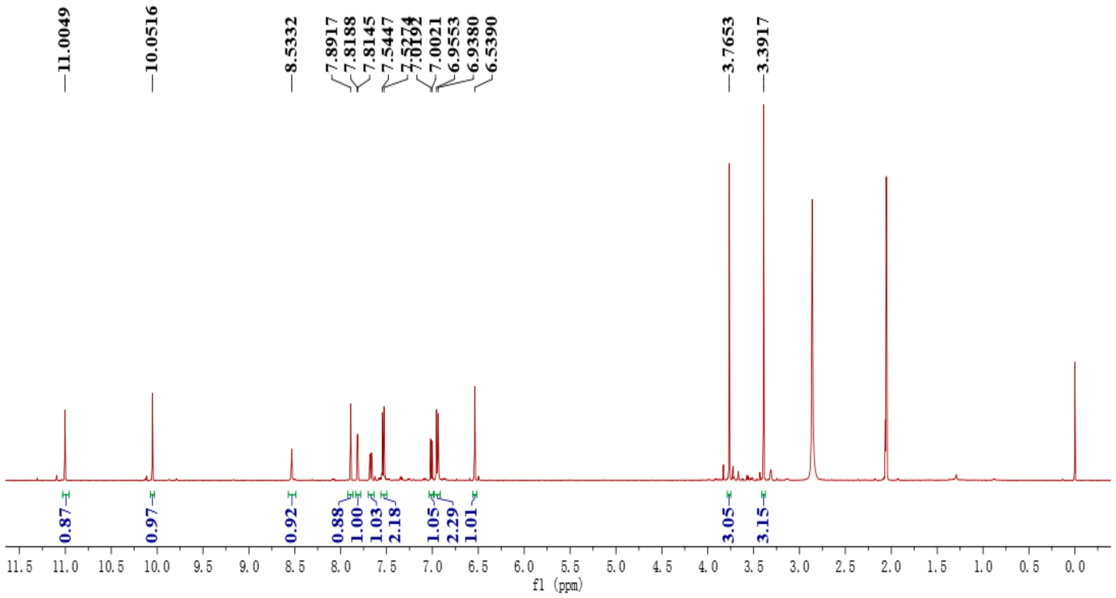

Figure S2. ^{13}C NMR (125 MHz, acetone-d_6) spectrum of compound **1.**

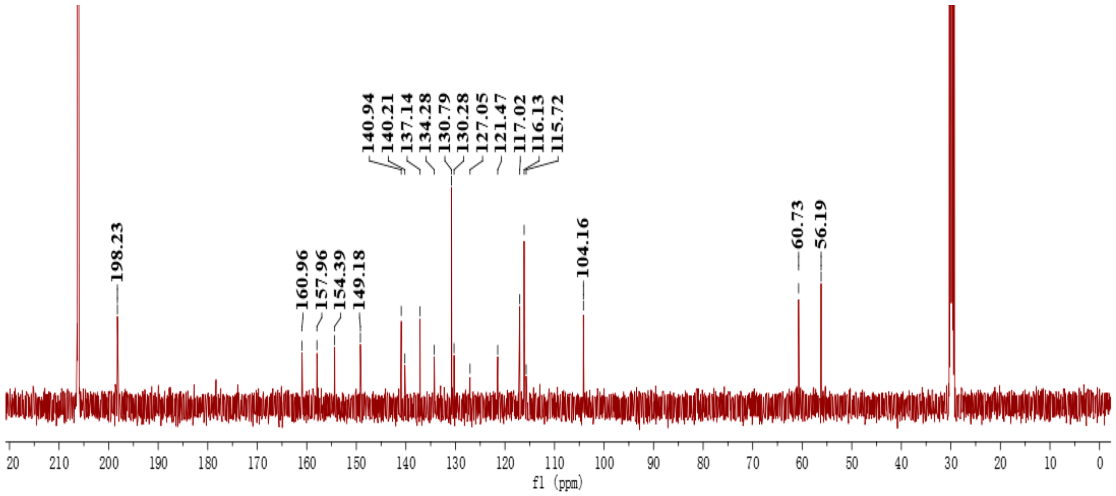

Figure S3. DEPT135 spectrum of compound **1.**

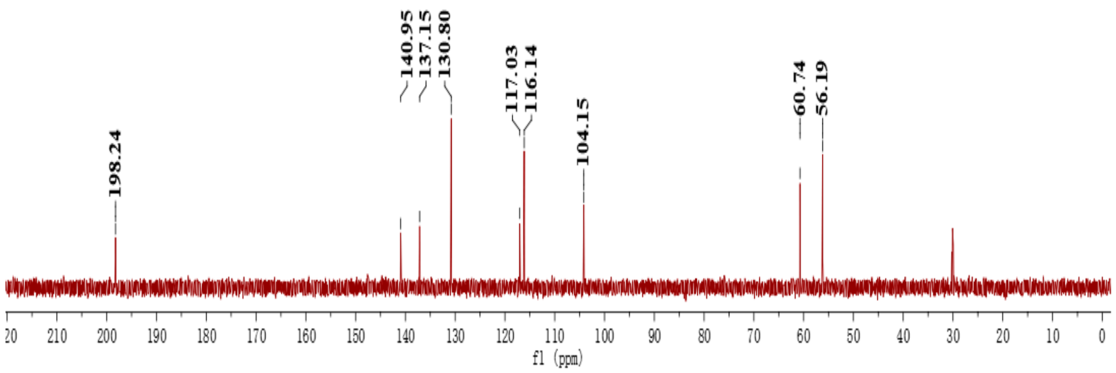

Figure S4. HSQC spectrum of compound **1.**

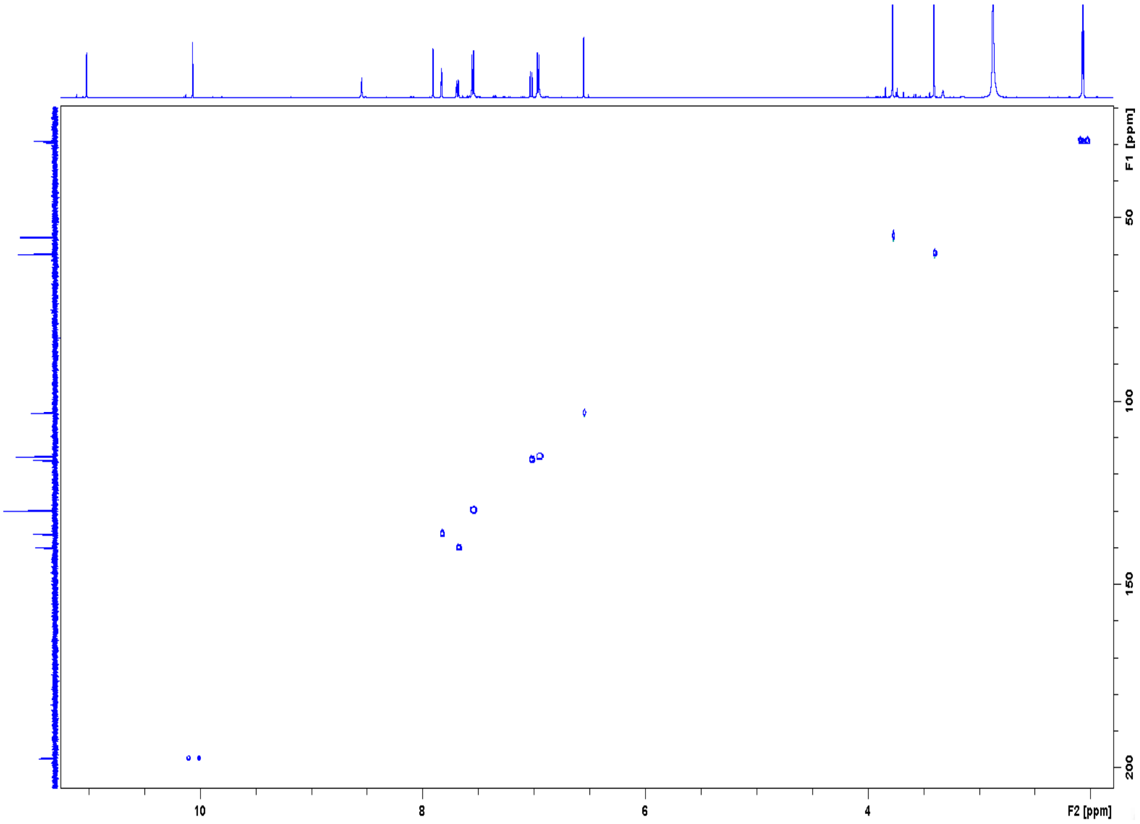

Figure S5. HMBC spectrum of compound **1.**

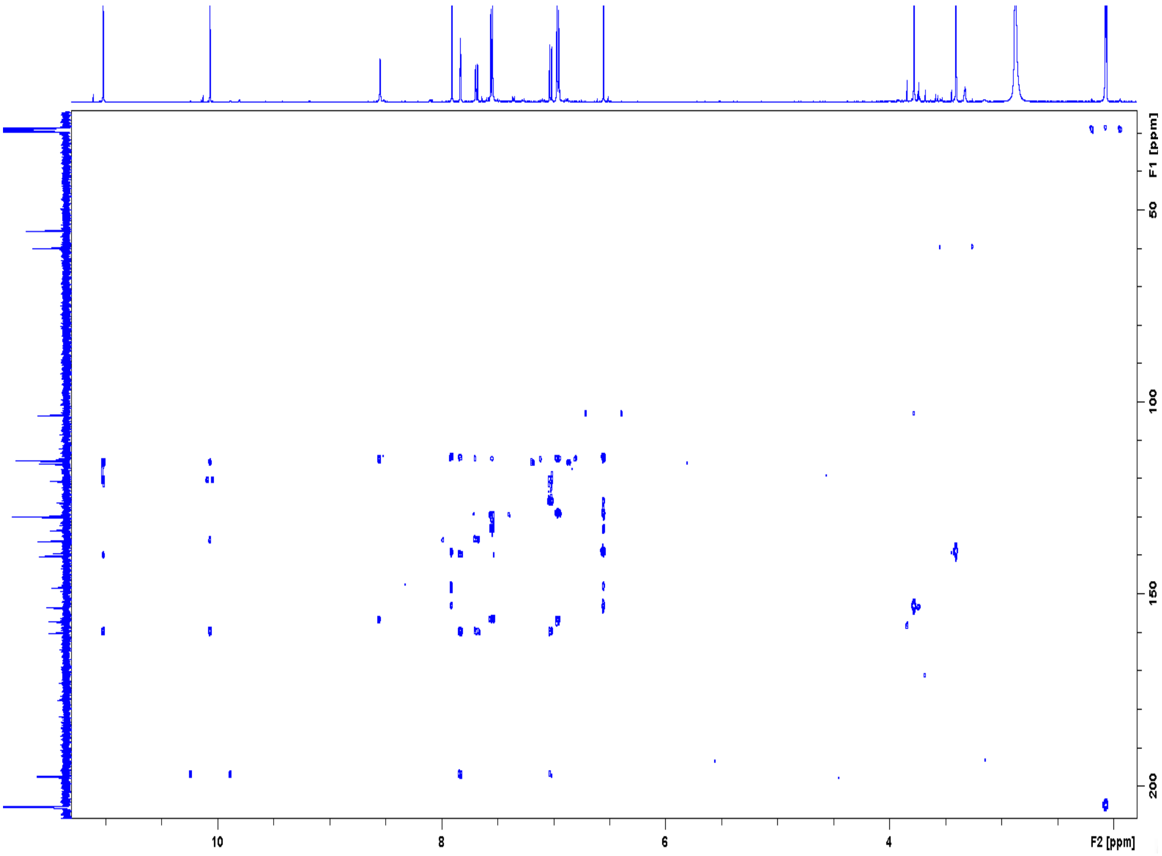

Figure S6. ^1H-^1H COSY spectrum of compound **1.**

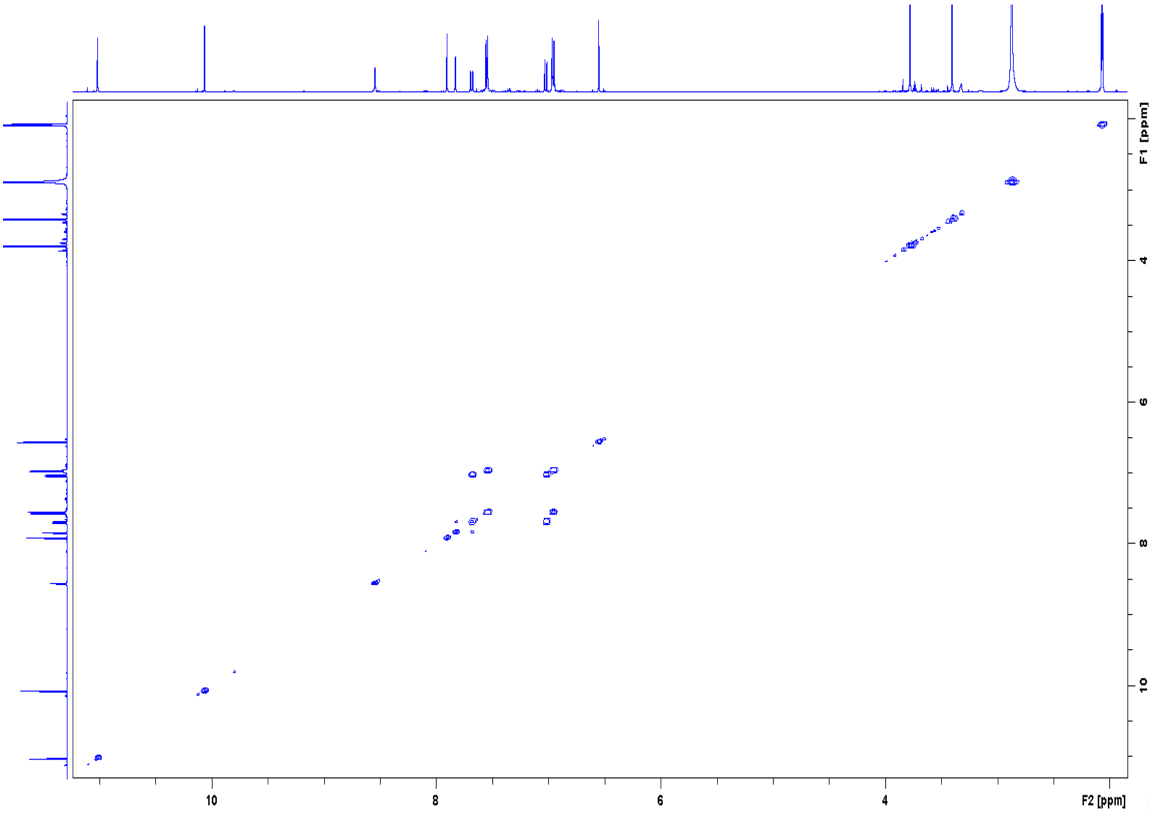

Figure S7. ROESY spectrum of compound **1.**

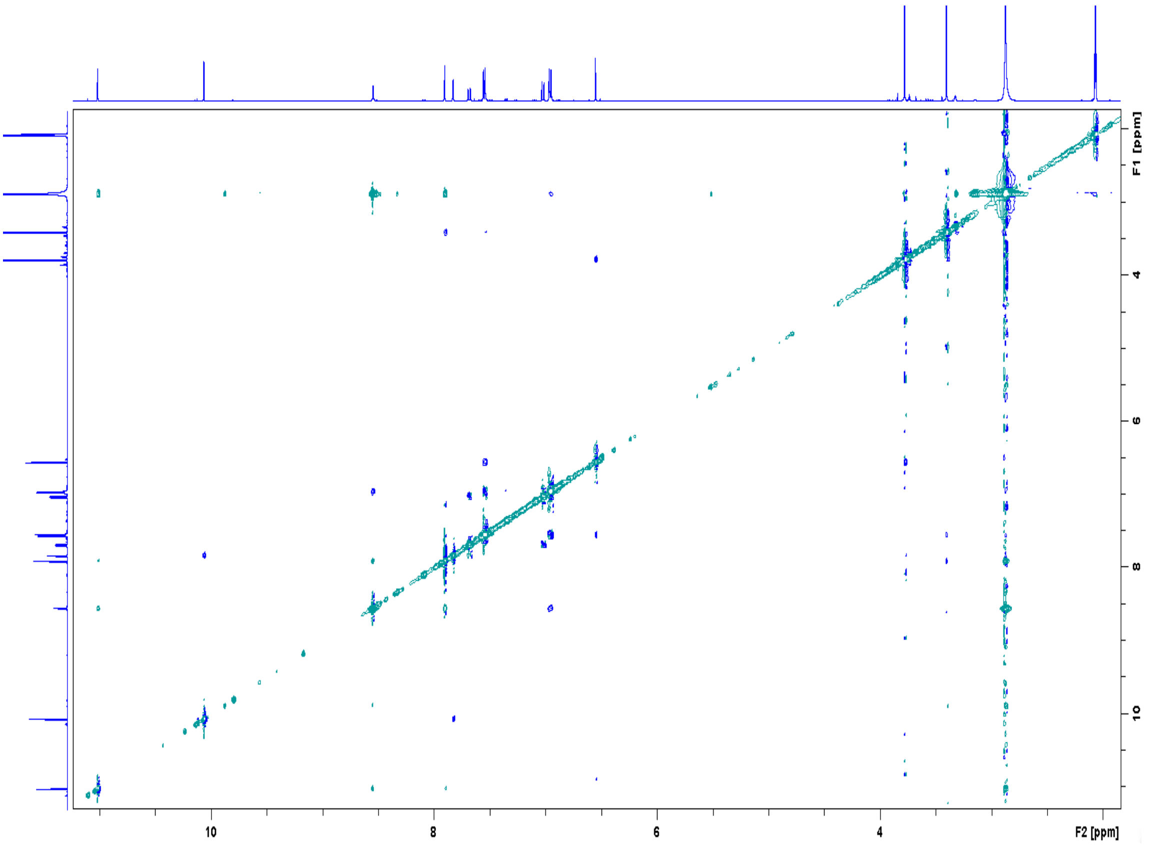

Figure S8. The HRESIMS spectrum of compound **1.**

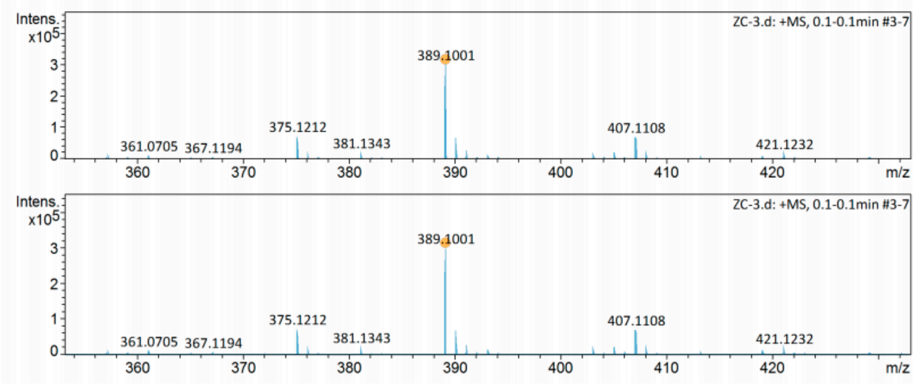

Meas. m/z	#	Ion Formula	m/z	err [ppm]	mSigma	# mSigma	Score	rdb	e$^-$ Conf	N-Rule	Adduct
389.1001	1	C21H18NaO6	389.0996	-1.5	29.1	1	100.00	13.0	even	ok	M+Na

Figure S9. UV spectrum of compound **1.**

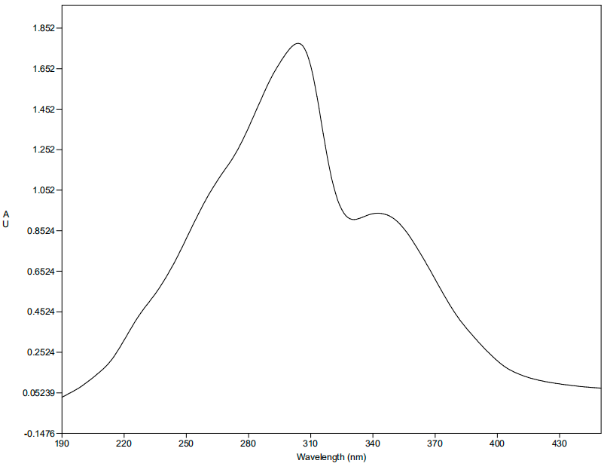

Bio-Kine Software V4.80 Date : 2022/10/22 Time : 21:29:43

COMMENTS :
File name : e:\2022年数据\郭志凯 课题组\zc\zc-3-uv.bka
MOS-500 Spectrometer
Spectrum measurement
Acq duration = 0.2 s
Fentes bandwidth = .5 nm
HV = 126 V
Biokine V4.80

Figure S10. IR spectrum of compound **1.**

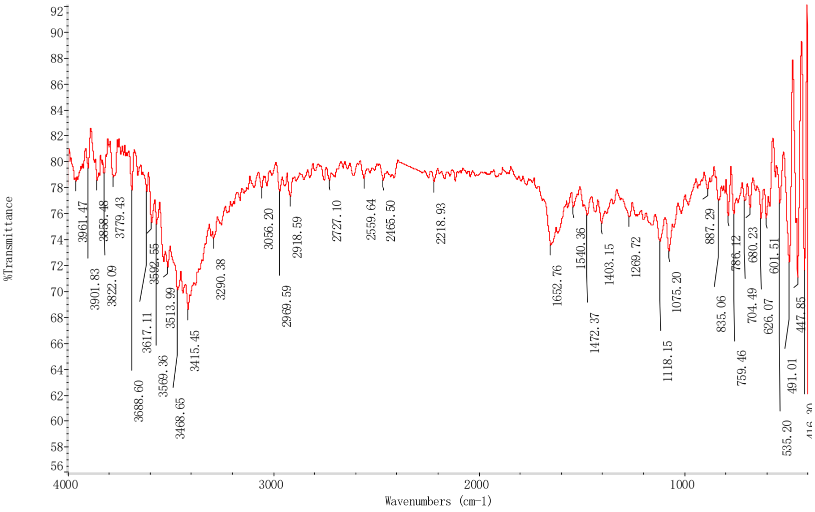

Figure S11. ^{1}H NMR (500 MHz, $CDCl_3$) spectrum of compound **2**.

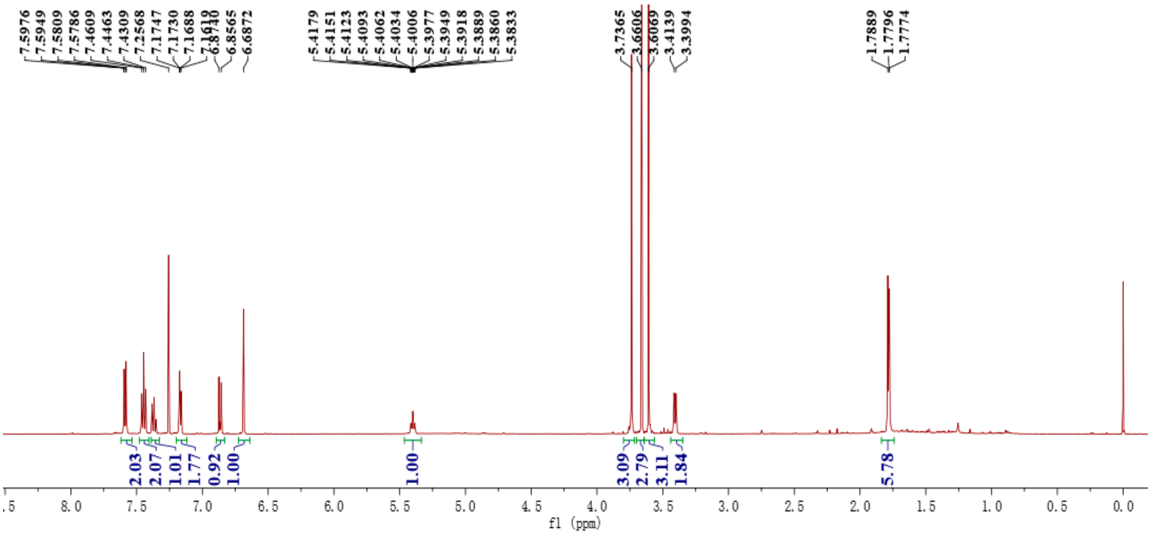

Figure S12. ^{13}C NMR (125 MHz, $CDCl_3$) spectrum of compound **2.**

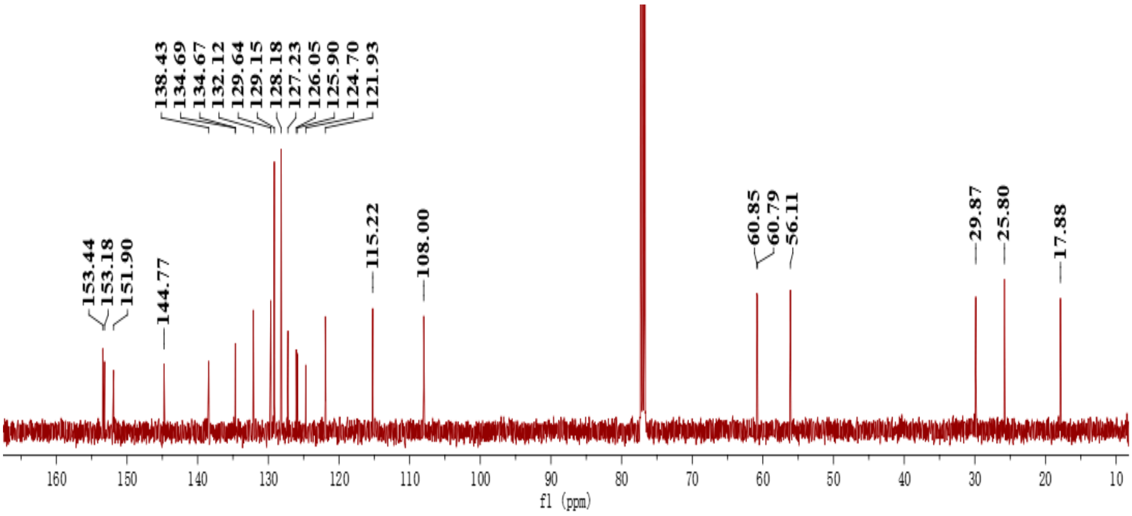

Figure S13. DEPT135 spectrum of compound **2**

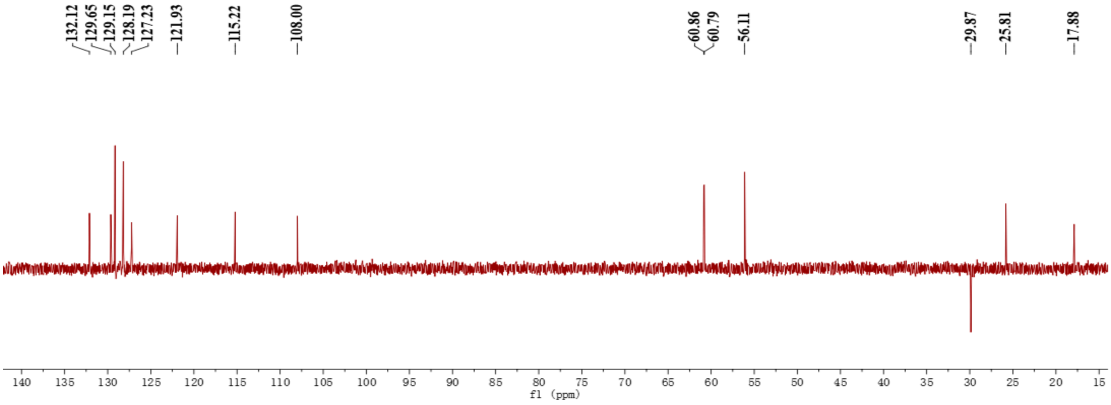

Figure S14. HSQC spectrum of compound **2.**

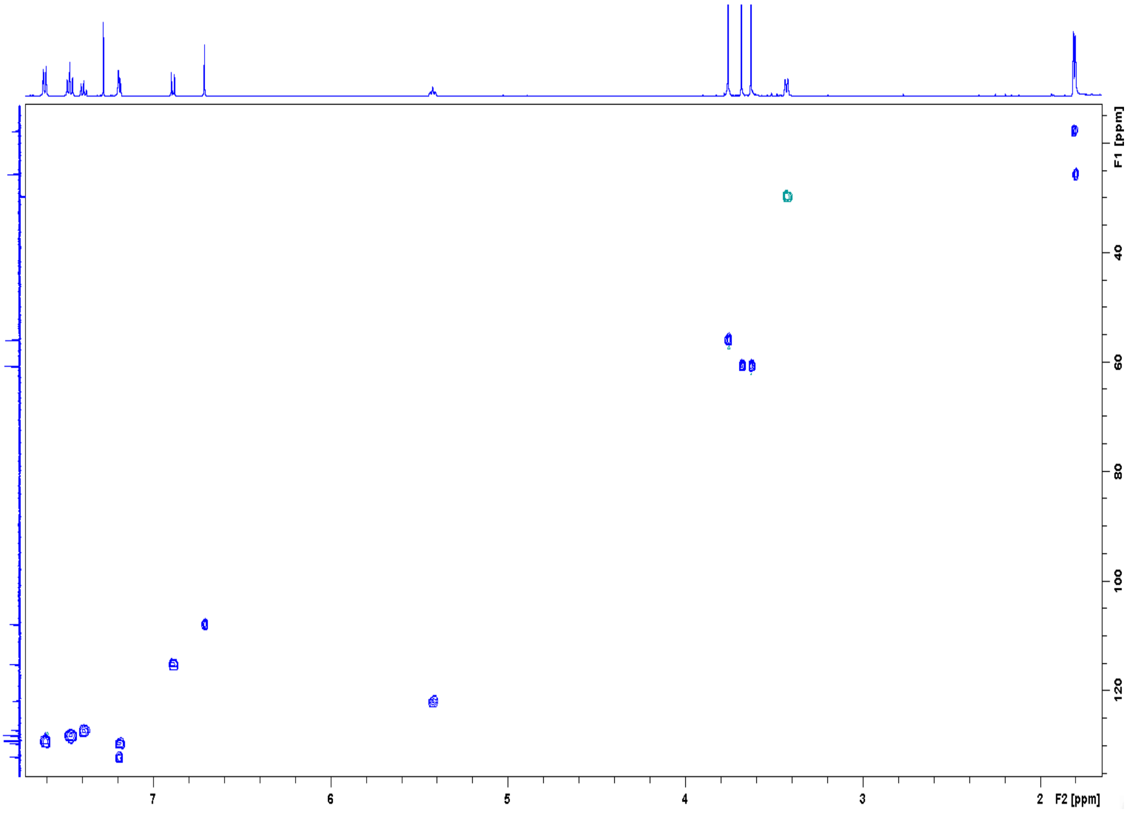

Figure S15. HMBC spectrum of compound **2.**

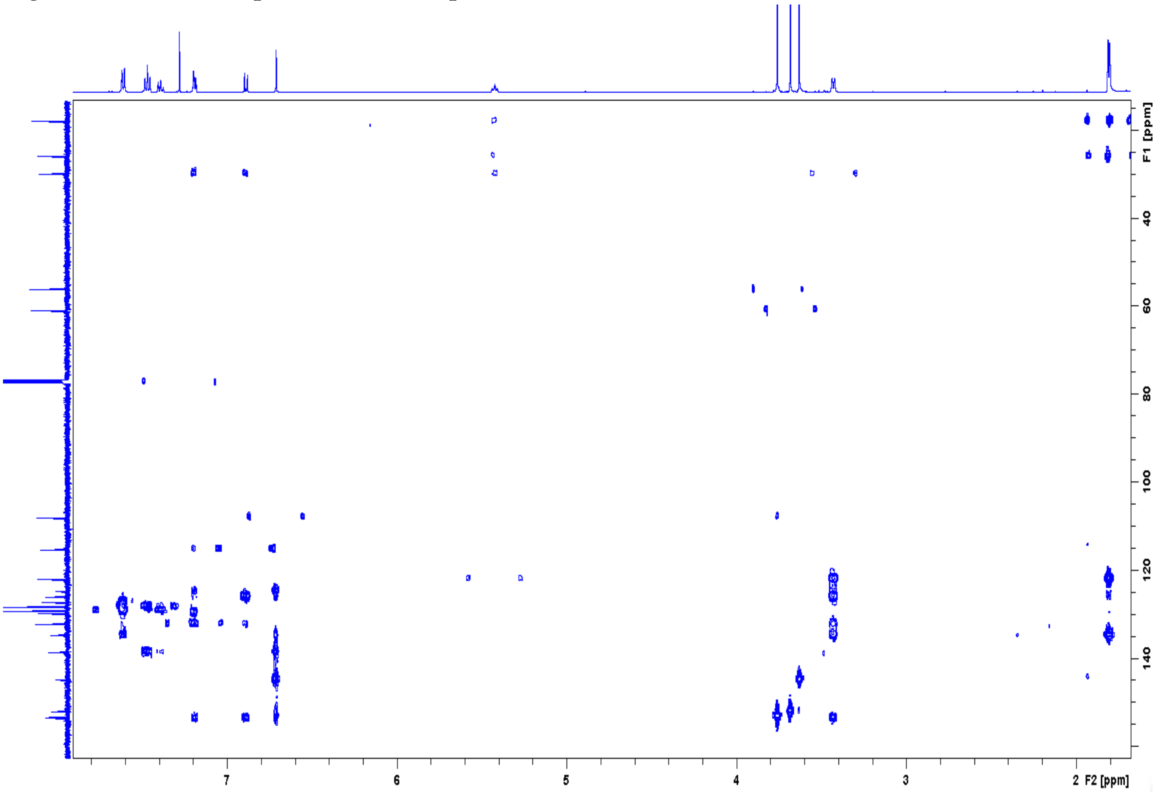

Figure S16. ^1H-^1H COSY spectrum of compound **2.**

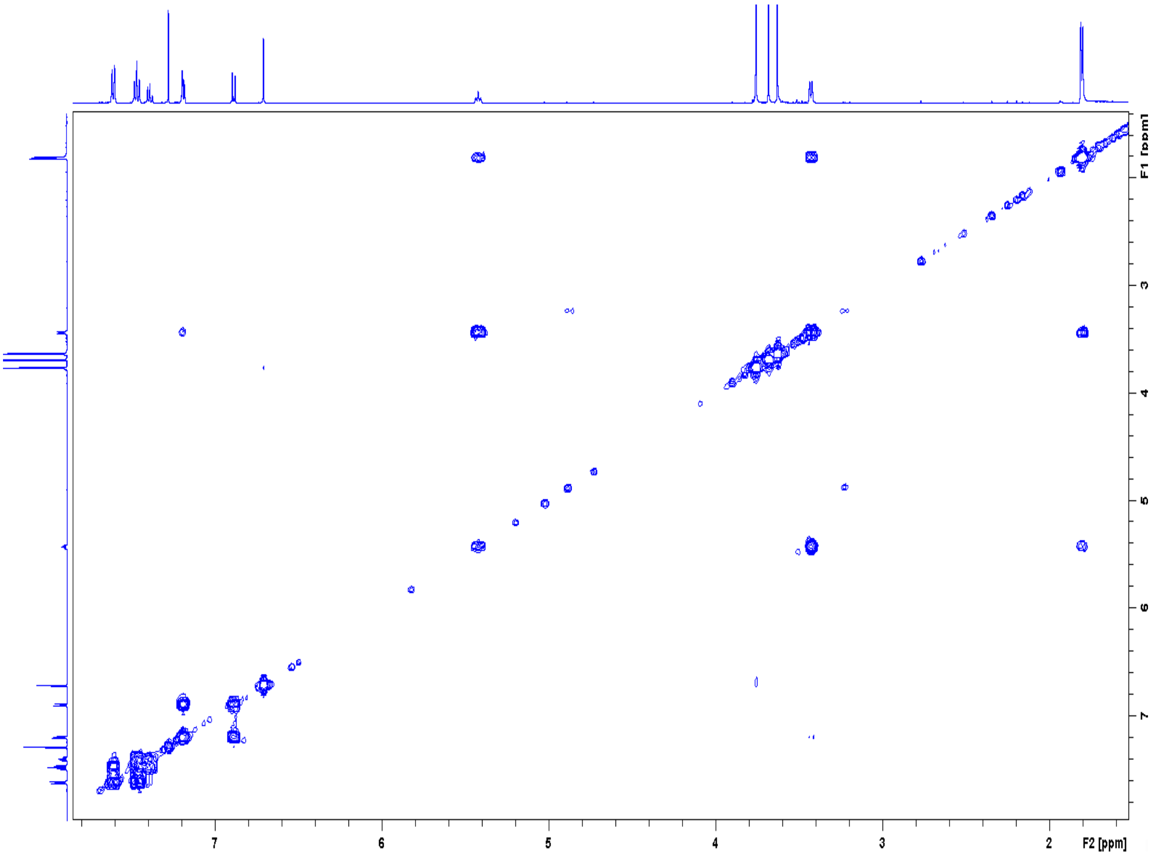

Figure S17. ROESY spectrum of compound **2.**

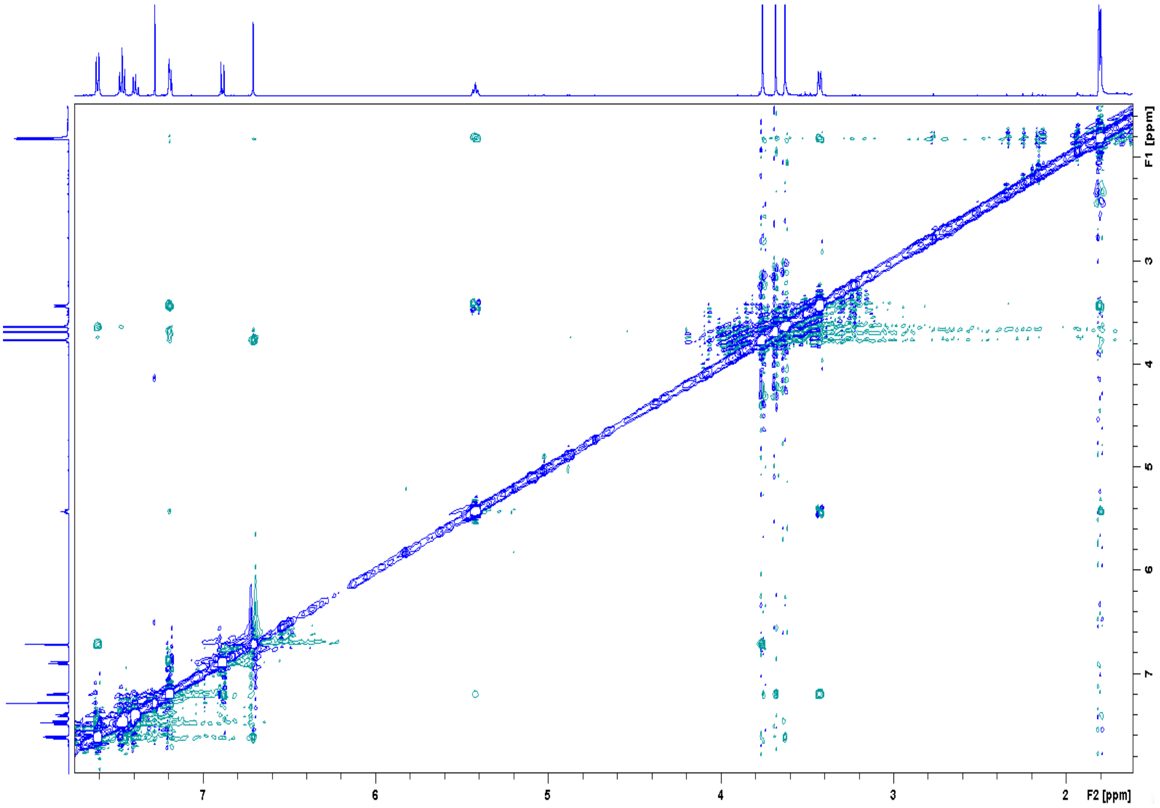

Figure S18. The HRESIMS spectrum of compound **2.**

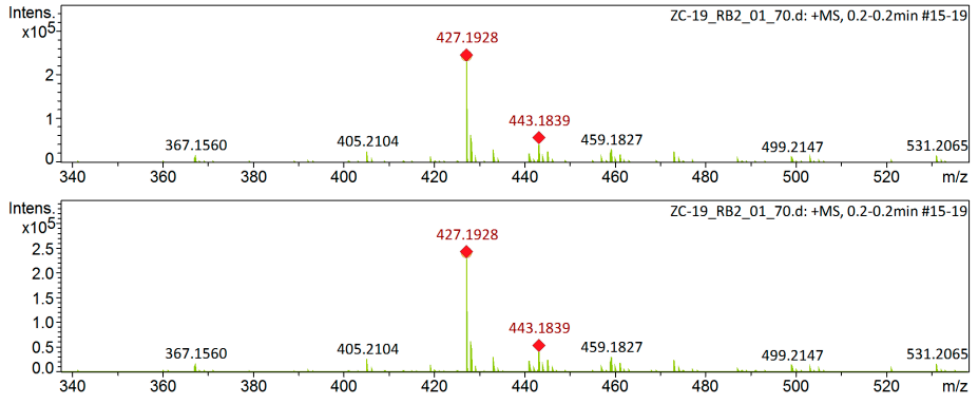

Meas. m/z	#	Ion Formula	m/z	err [ppm]	mSigma	# mSigma	Score	rdb	e⁻ Conf	N-Rule	Adduct
427.1928	1	C26H28NaO4	427.1880	-11.2	7.1	1	100.00	13.0	even	ok	M+Na

Figure S19. UV spectrum of compound **2.**

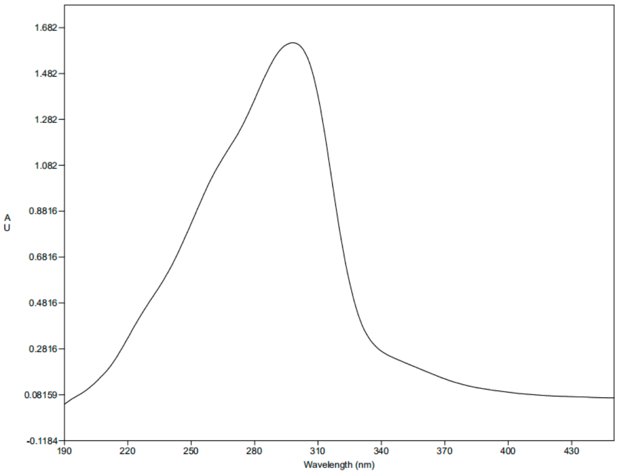

Bio-Kine Software V4.80 Date : 2022/10/22 Time : 21:52:49

COMMENTS :
File name : e:\2022年数据\郭志凯 课题组\zc\zc-19-uv.bka
MOS-500 Spectrometer
Spectrum measurement
Acq duration = 0.2 s
Fentes bandwidth = .5 nm
HV = 126 V
Biokine V4.80

Figure S20. IR spectrum of compound **2.**

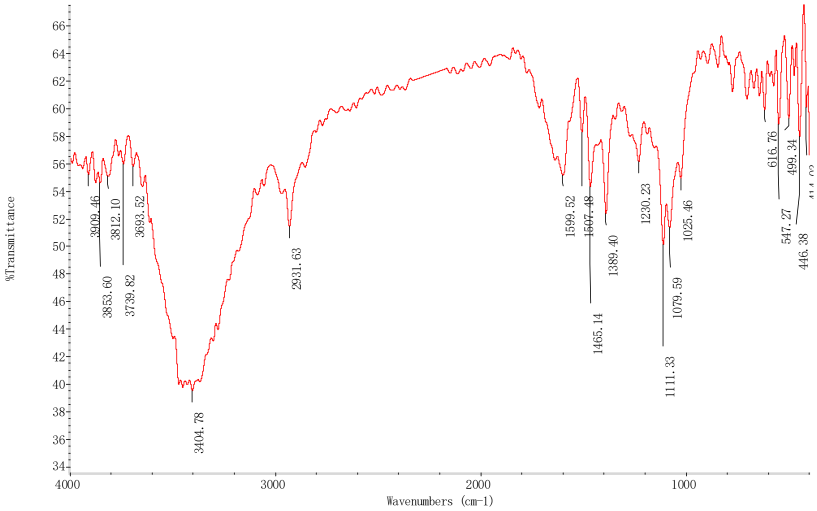

Figure S21. ^{1}H NMR (500 MHz, $CDCl_3$) spectrum of compound **3.**

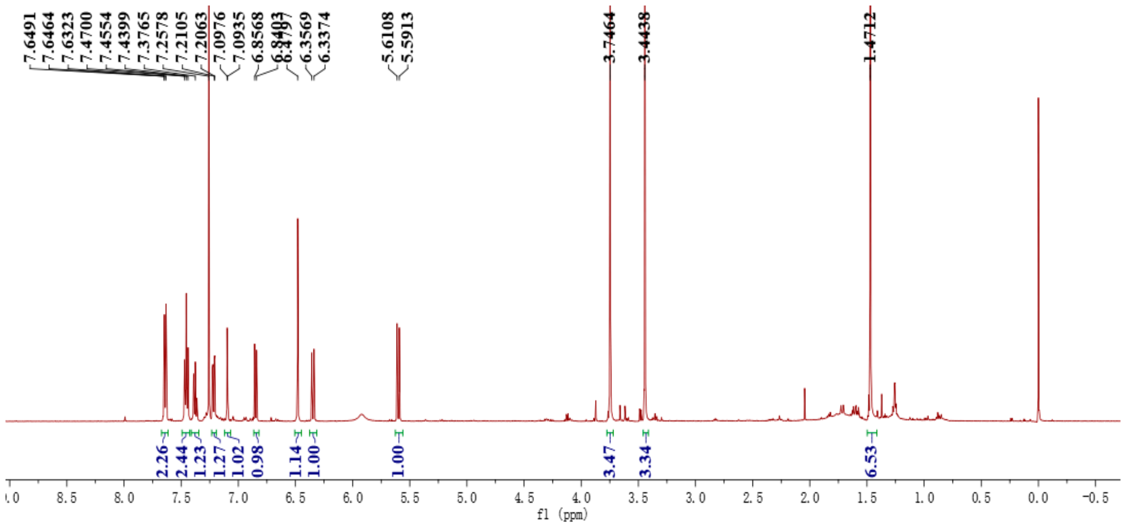

Figure S22. ^{13}C NMR (125 MHz, $CDCl_3$) spectrum of compound **3.**

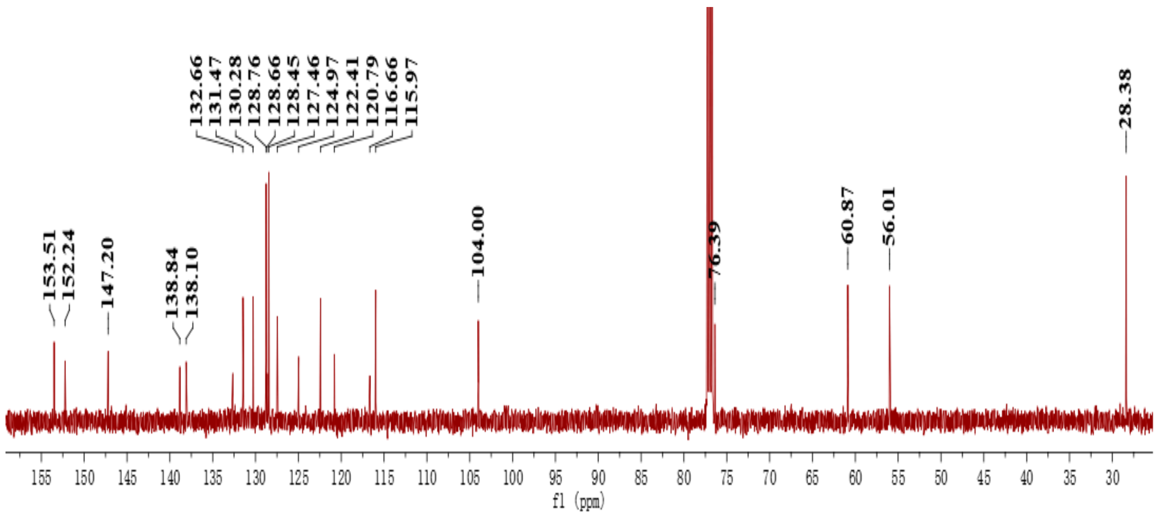

Figure S23. DEPT135 spectrum of compound **3.**

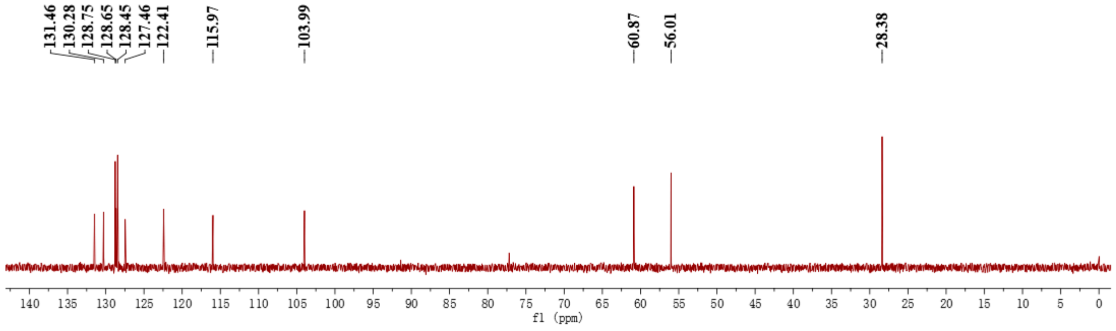

Figure S24. HSQC spectrum of compound **3.**

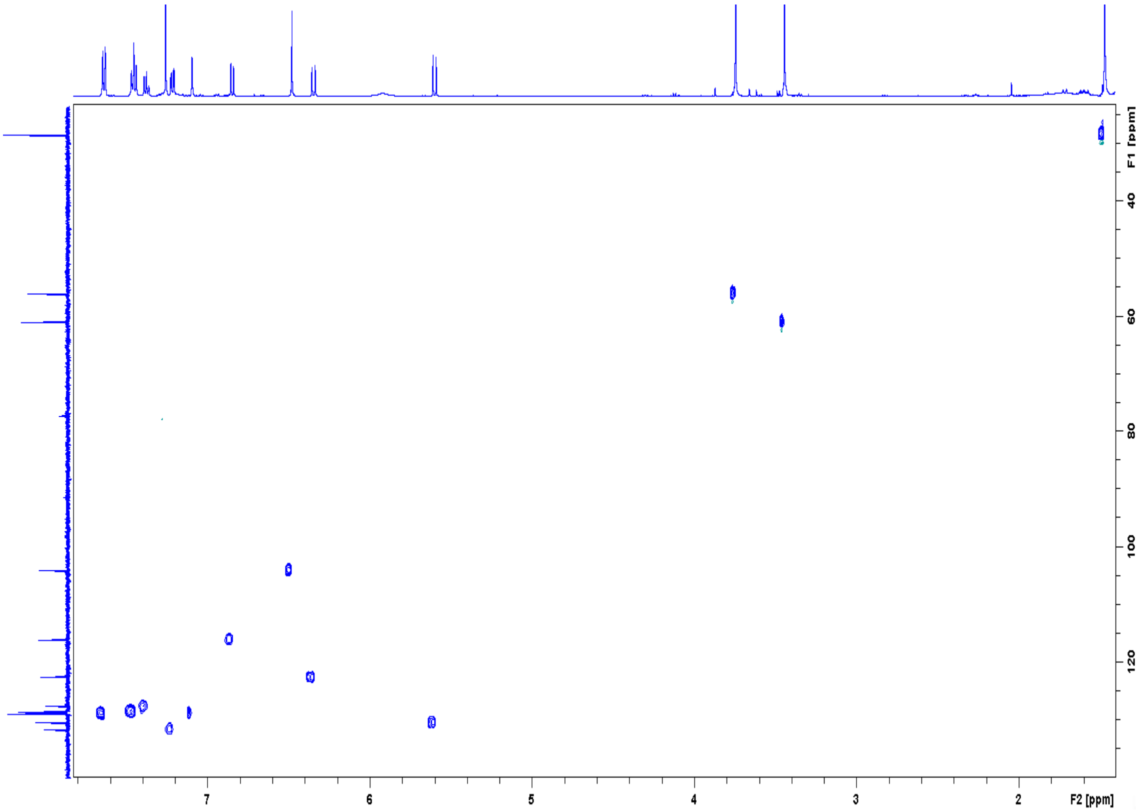

Figure S25. HMBC spectrum of compound **3.**

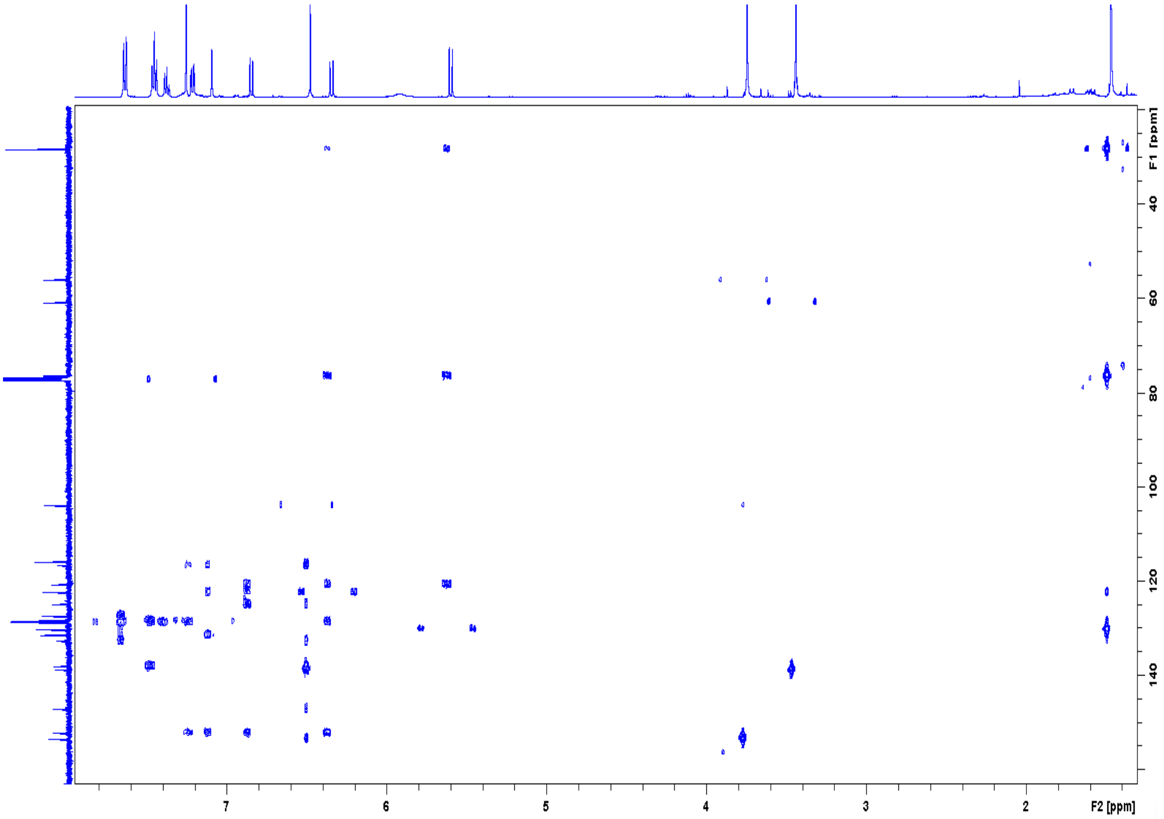

Figure S26. ^{1}H-^{1}H COSY spectrum of compound **3.**

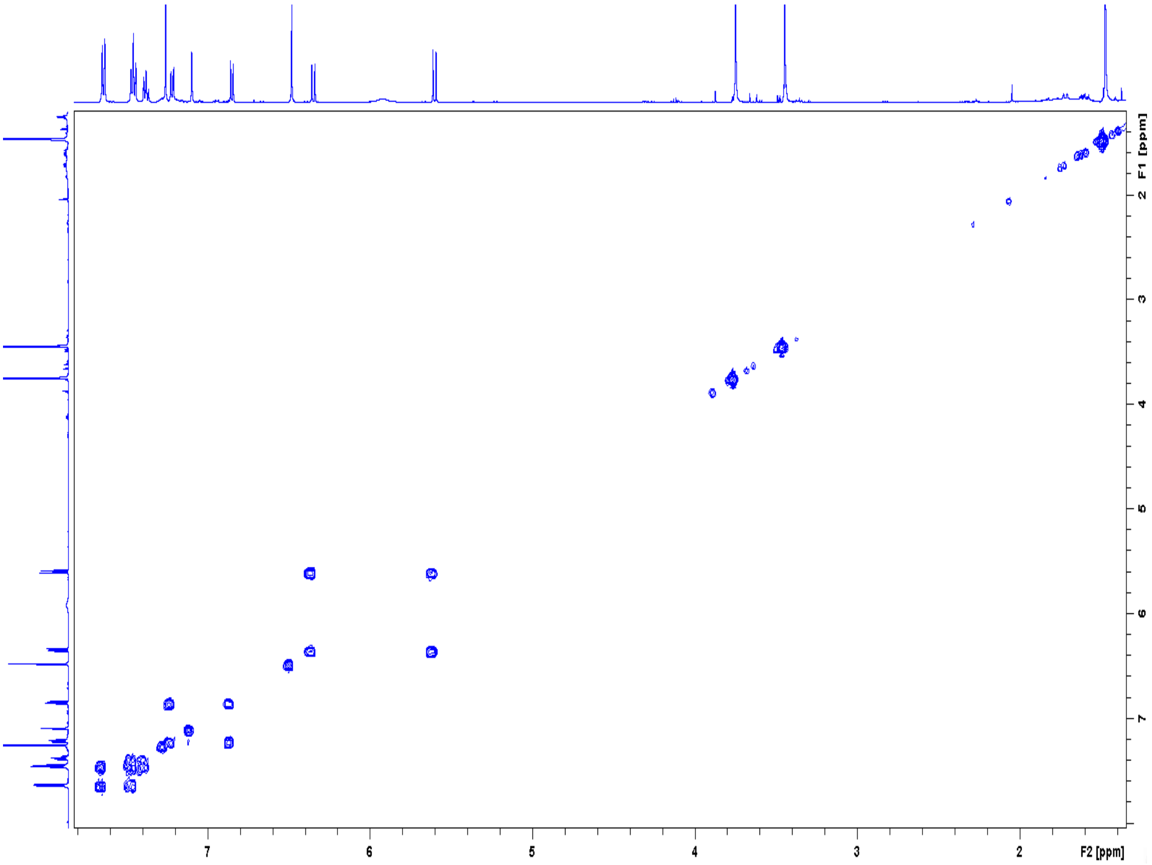

Figure S27. ROESY spectrum of compound **3.**

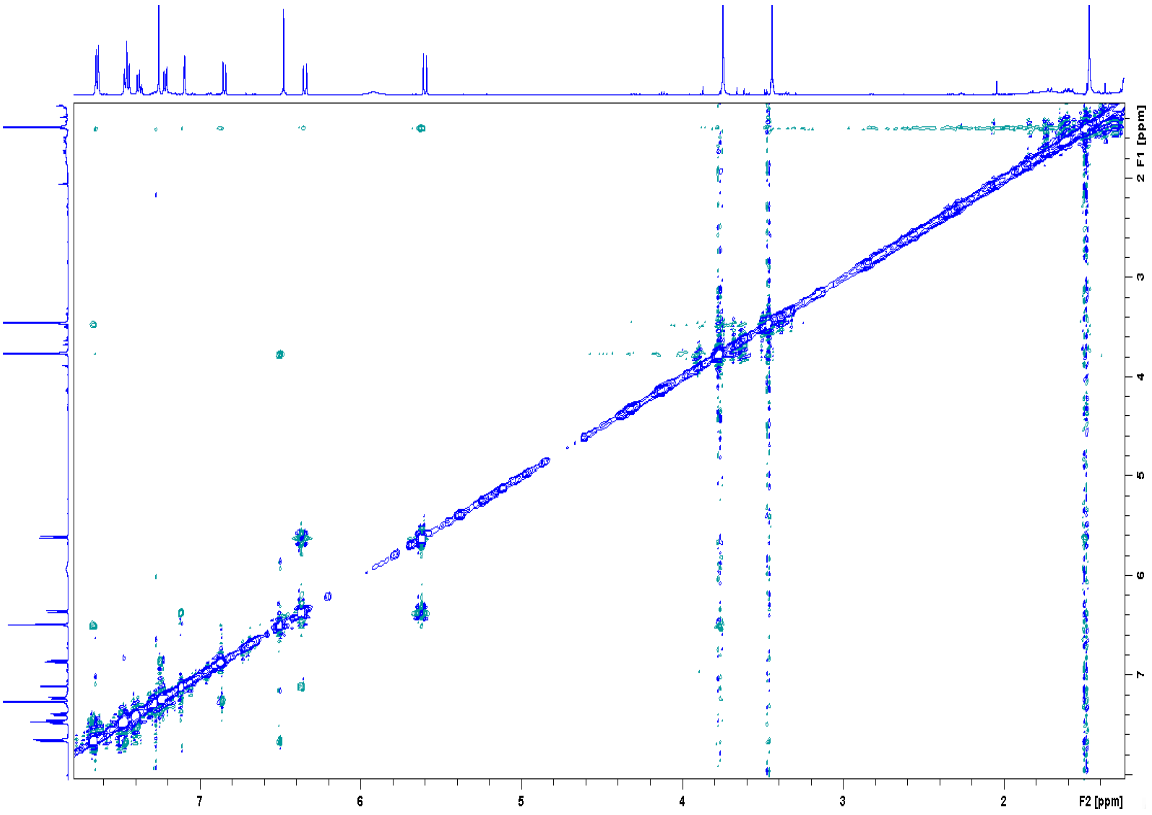

Figure S28. The HRESIMS spectrum of compound **3.**

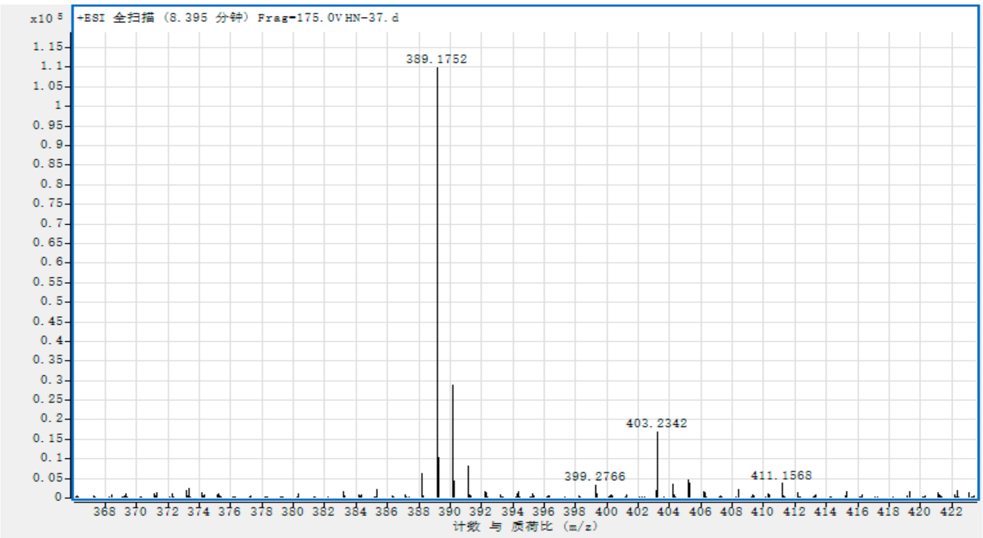

Figure S29. UV spectrum of compound **3.**

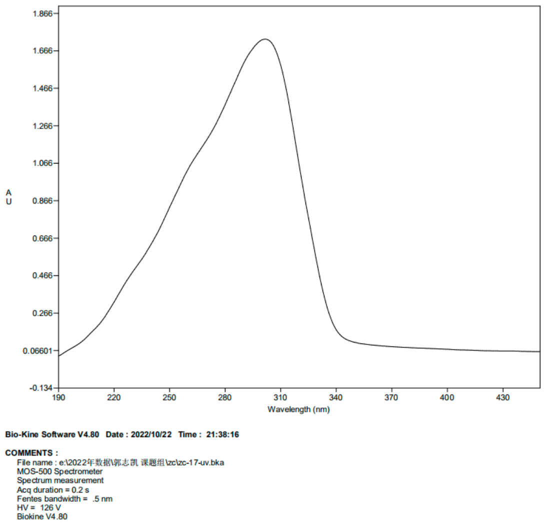

Figure S30. IR spectrum of compound **3.**

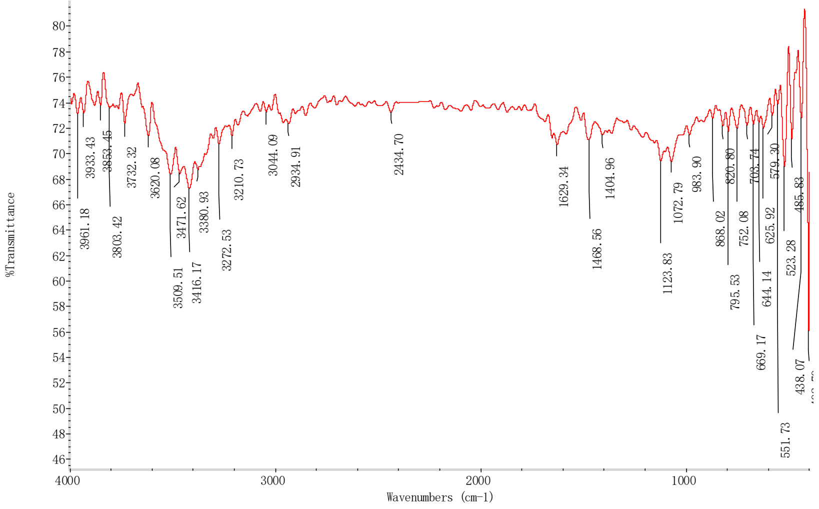

Figure S31. ^{1}H NMR (500 MHz, CDCl3) spectrum of compound **4.**

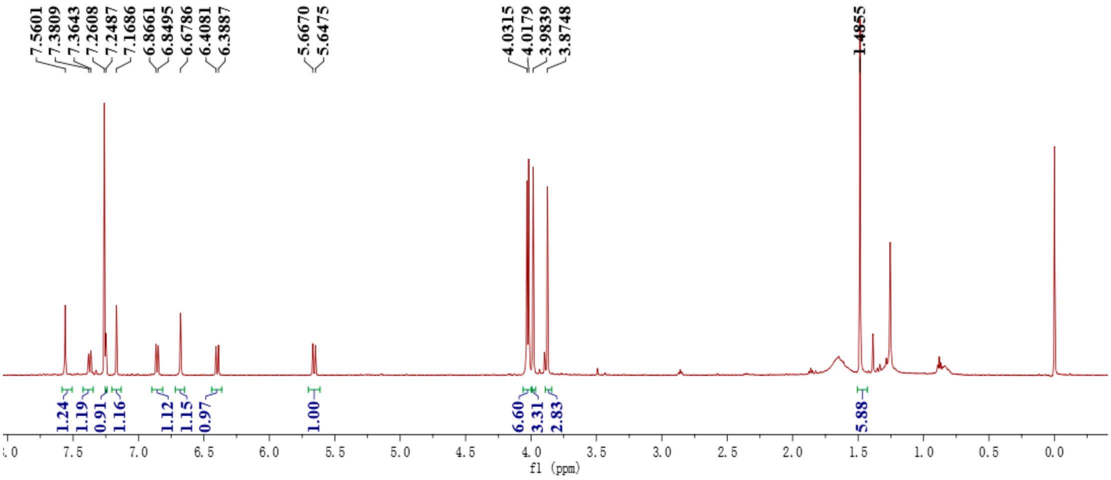

Figure S32. ^{13}C NMR (125 MHz, CDCl3) spectrum of compound **4.**

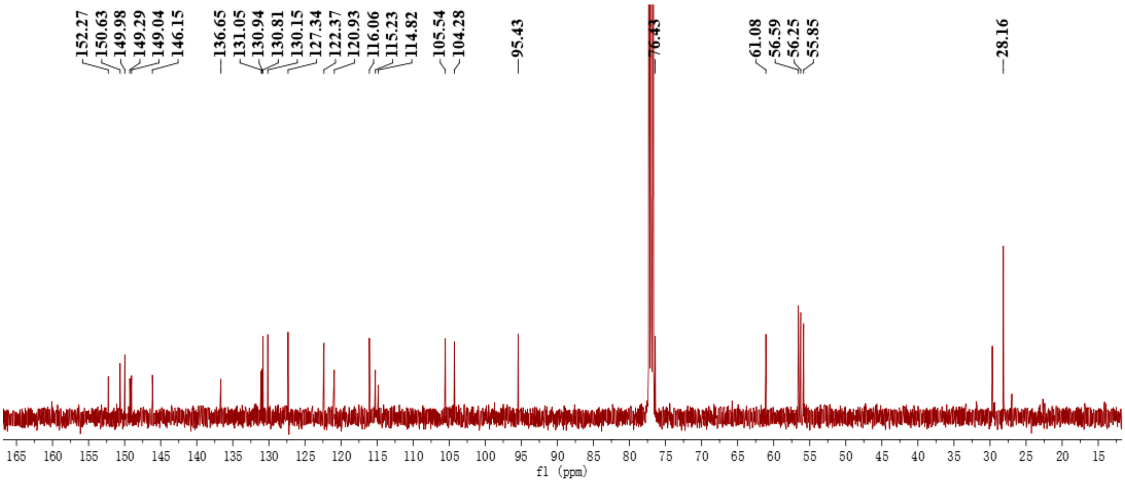

Figure S33. DEPT135 spectrum of compound **4.**

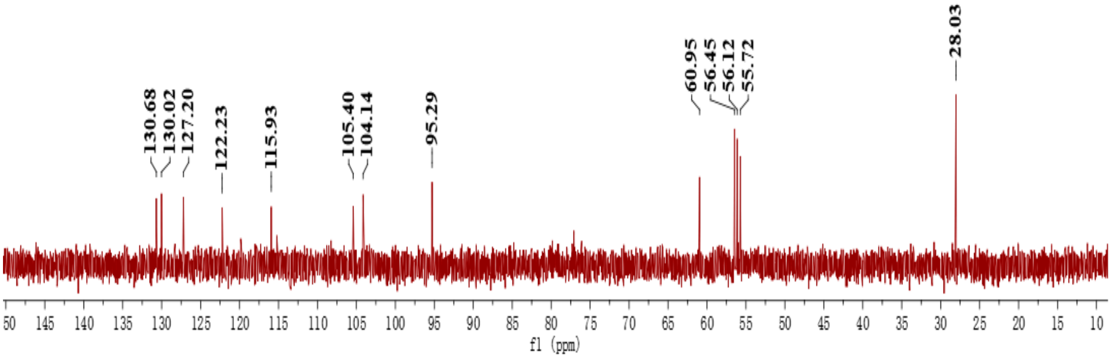

Figure S34. HSQC spectrum of compound **4.**

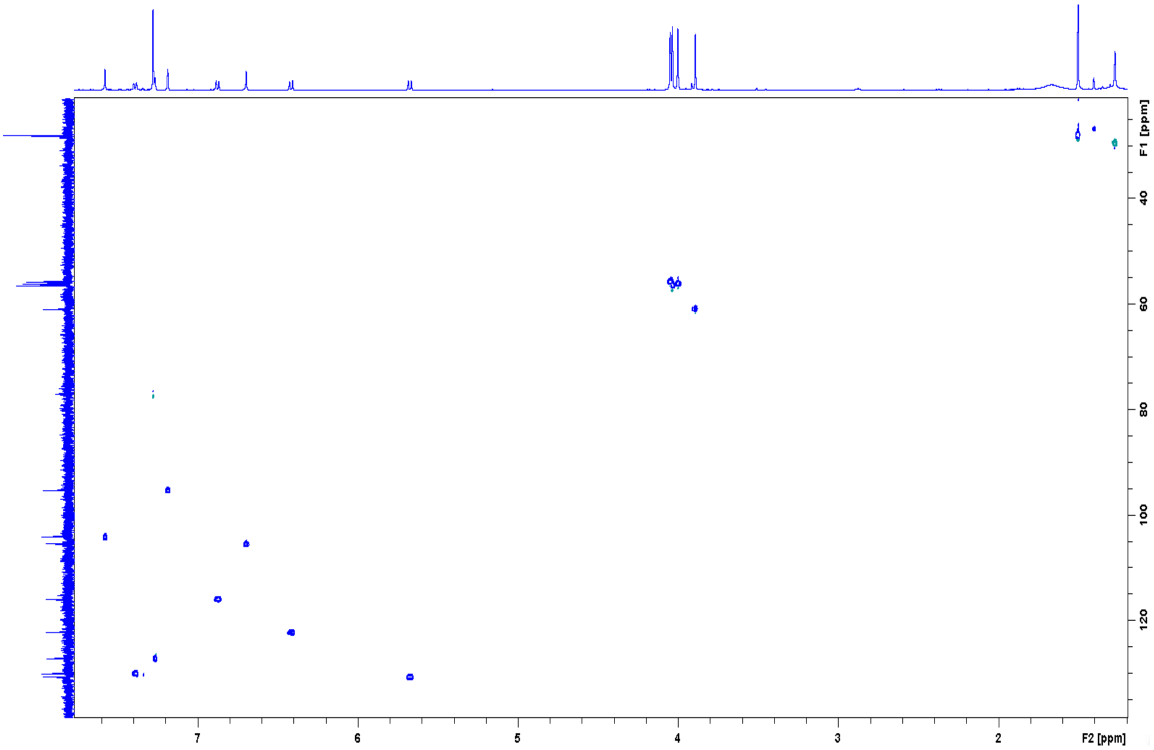

Figure S35. HMBC spectrum of compound **4.**

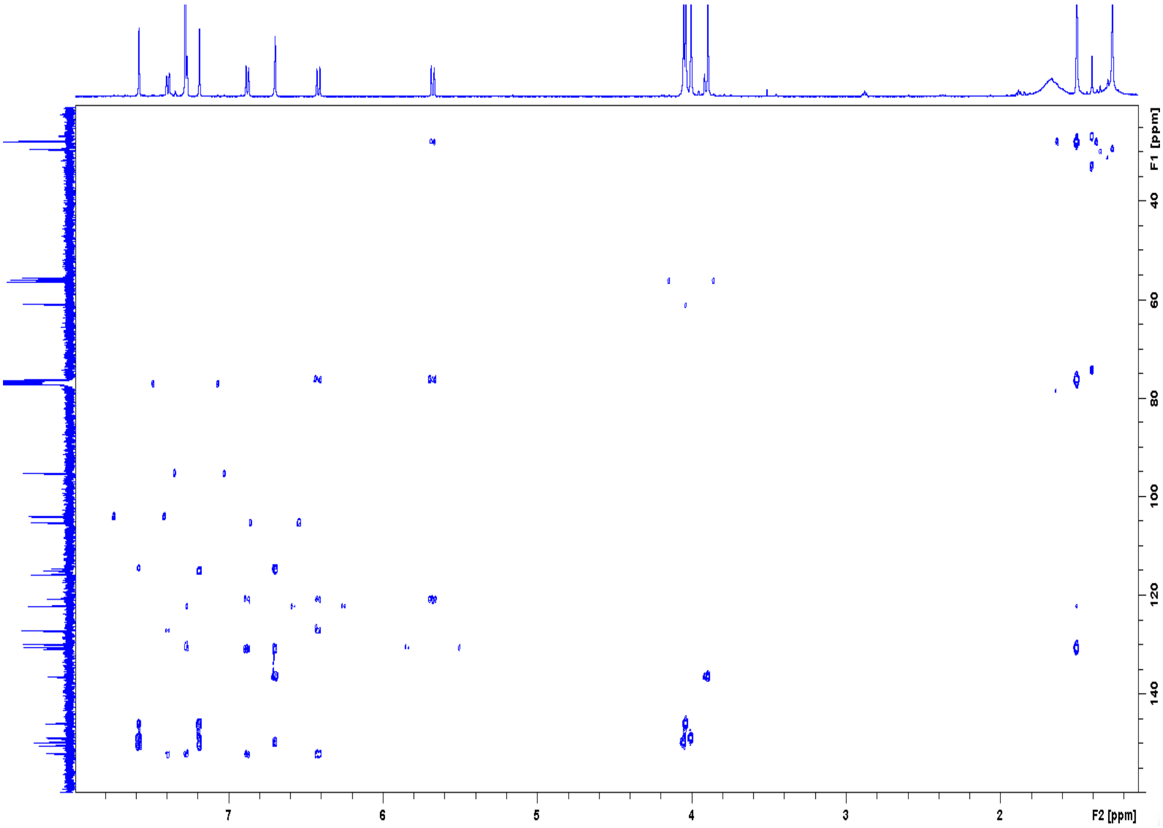

Figure S36. ^{1}H-^{1}H COSY spectrum of compound **4.**

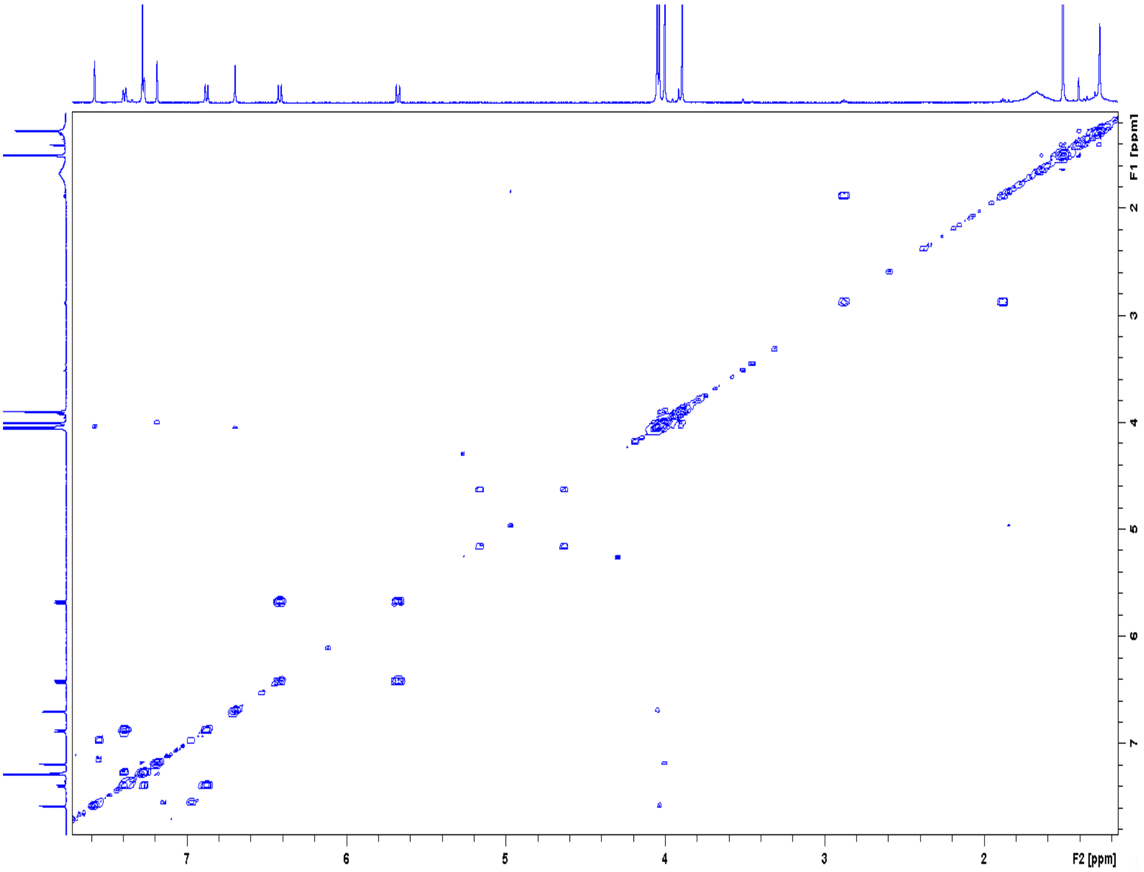

Figure S37. ROESY spectrum of compound **4.**

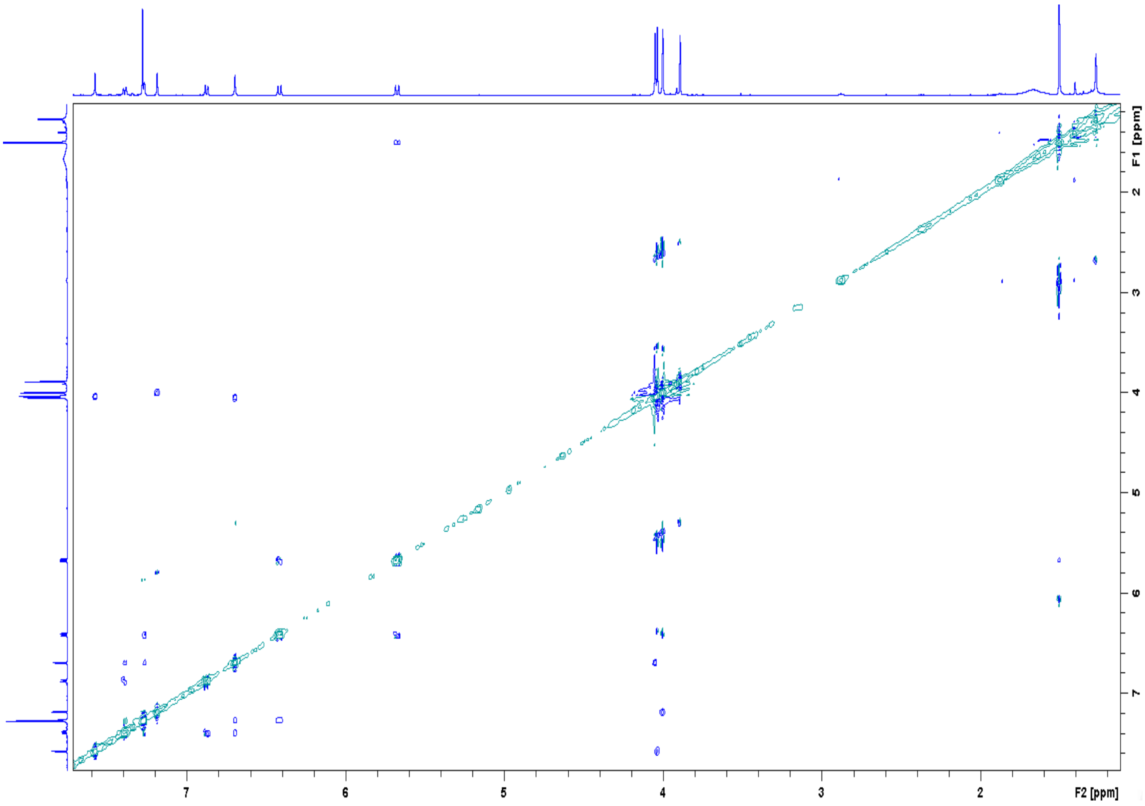

Figure S38. The HRESIMS spectrum of compound **4.**

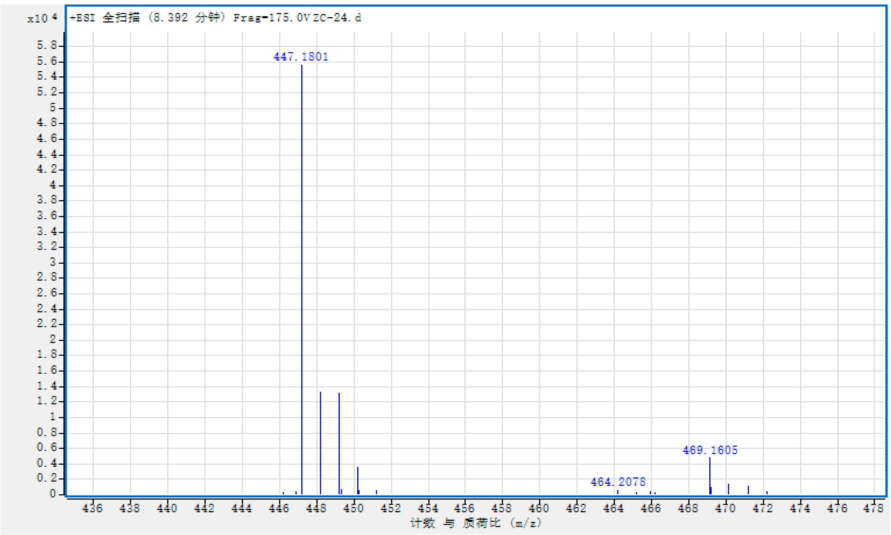

Figure S39. UV spectrum of compound **4.**

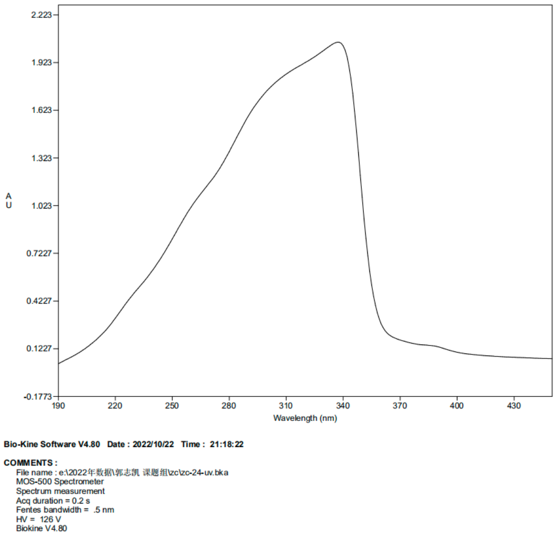

Figure S40. IR spectrum of compound **4.**

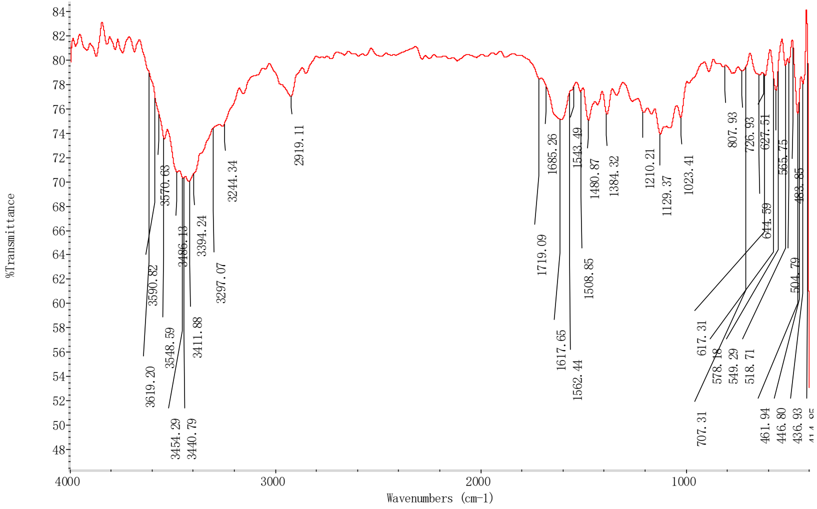

Figure S41. ^{1}H NMR (500 MHz, $CDCl_3$) spectrum of compound **5**.

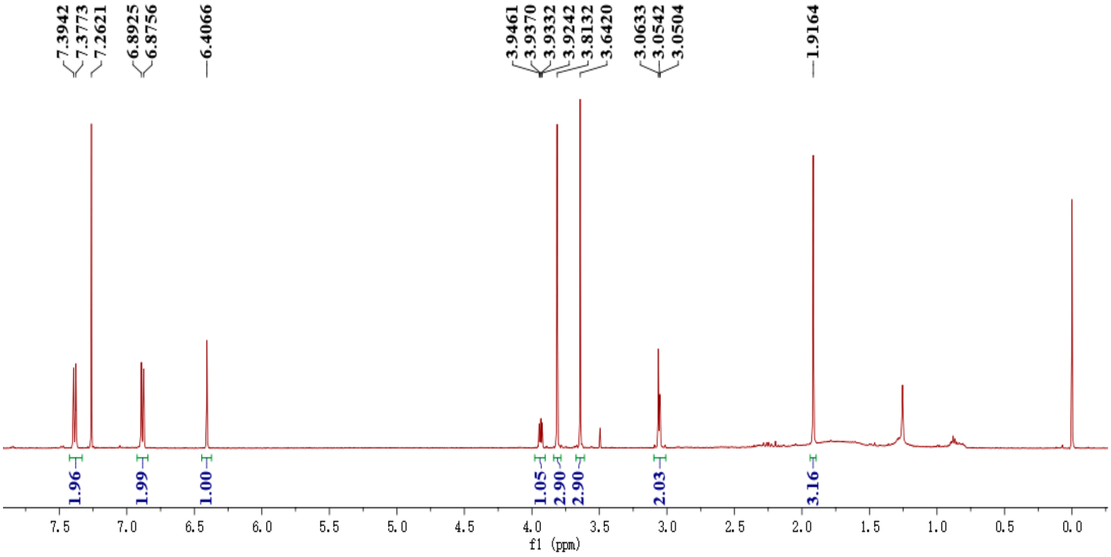

Figure S42. ^{13}C NMR (125 MHz, $CDCl_3$) spectrum of compound **5.**

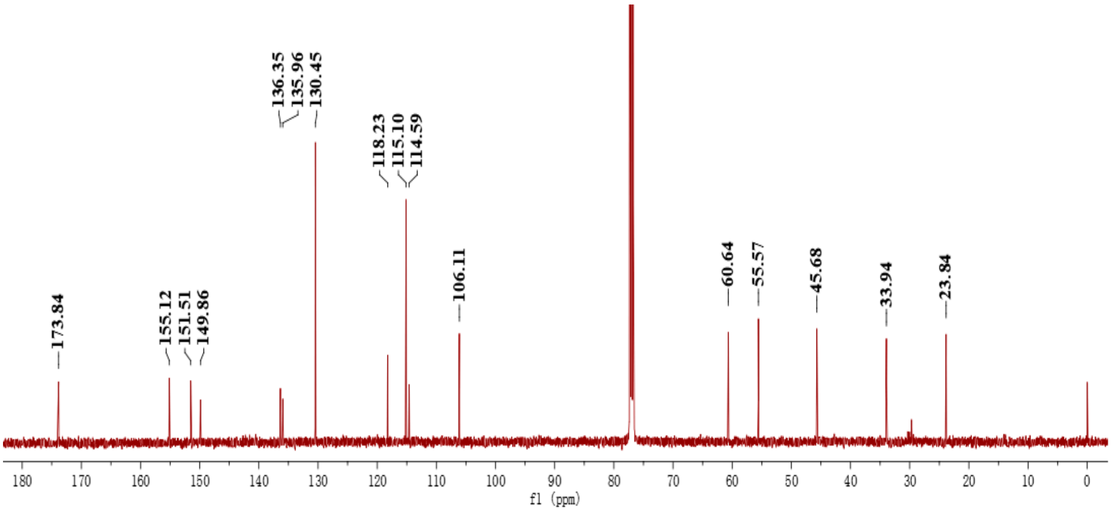

Figure S43. DEPT135 spectrum of compound **5.**

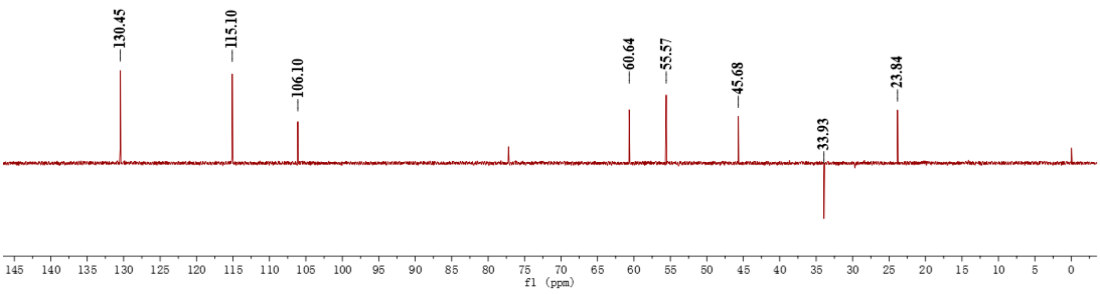

Figure S44. HSQC spectrum of compound **5.**

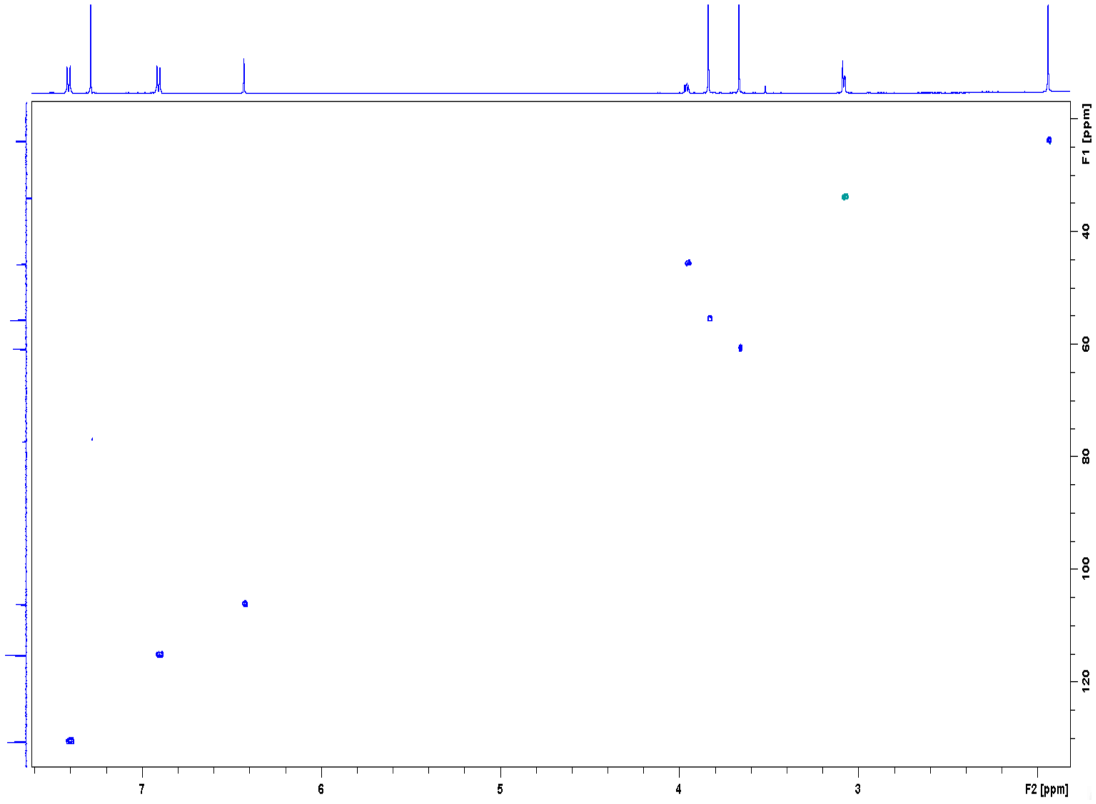

Figure S45. HMBC spectrum of compound **5.**

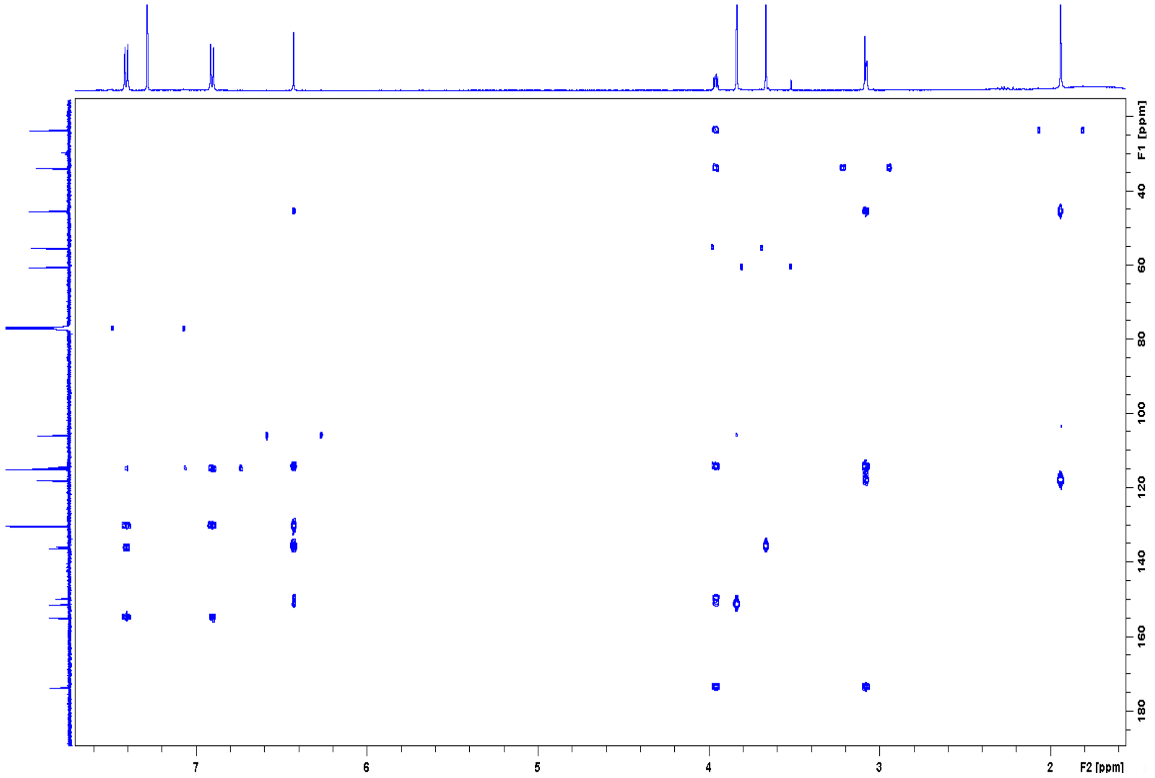

Figure S46. ^{1}H-^{1}H COSY spectrum of compound **5.**

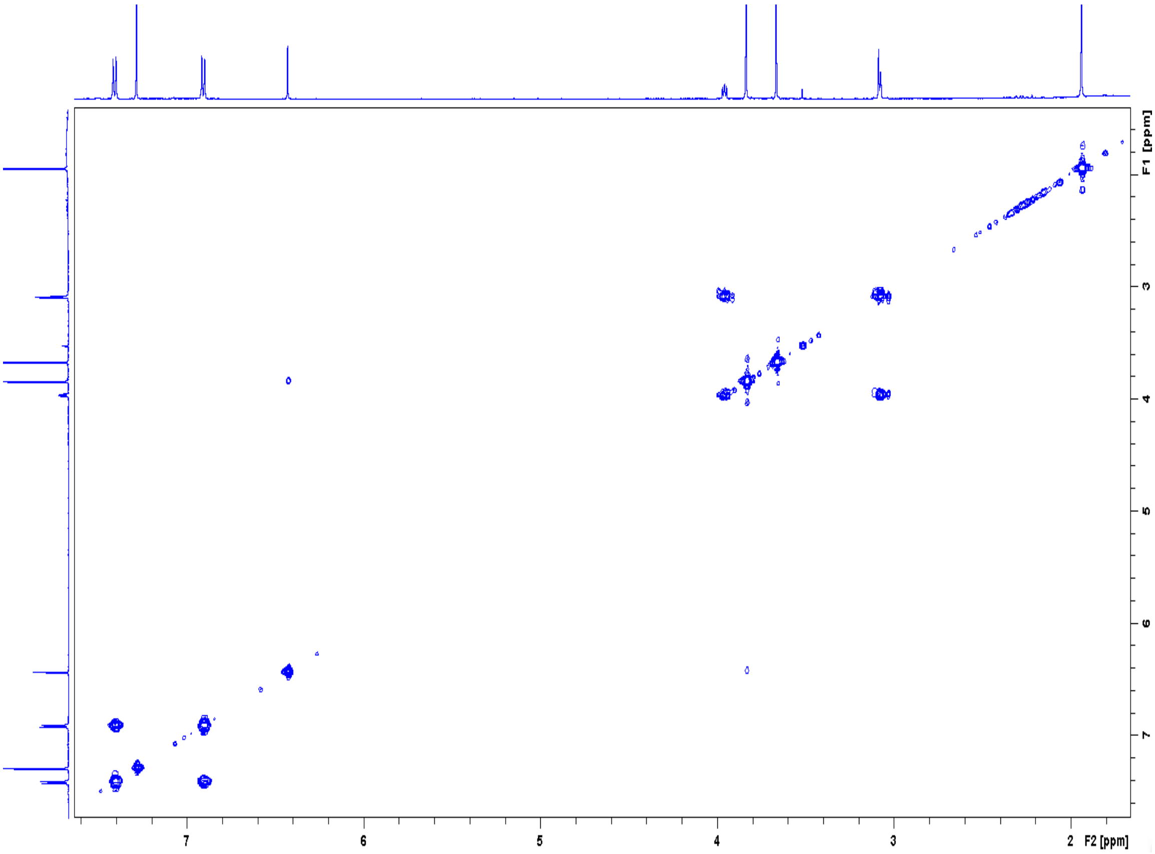

Figure S47. NOESY spectrum of compound **5.**

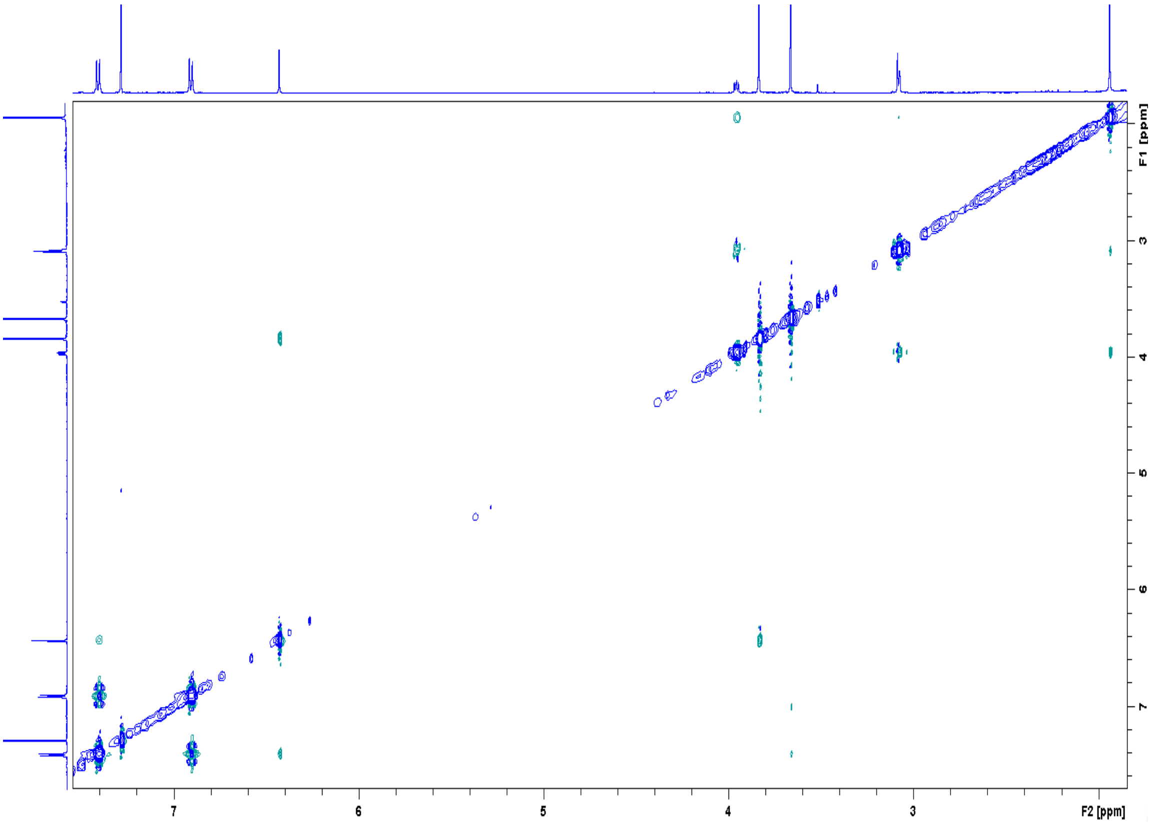

Figure S48. The HRESIMS spectrum of compound **5.**

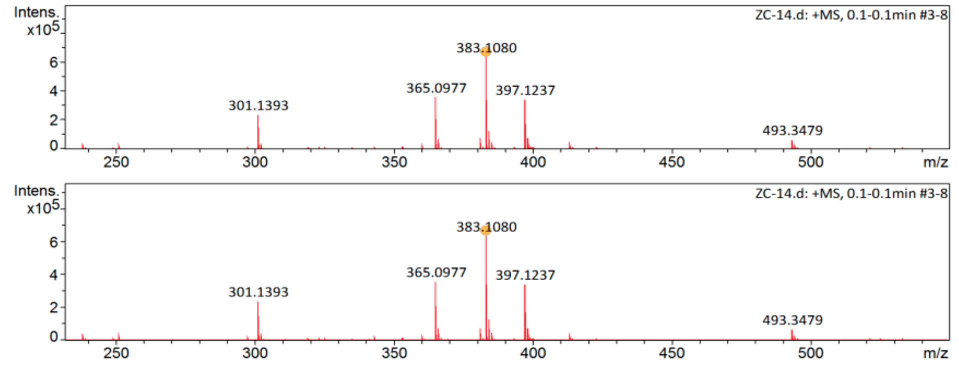

Meas. m/z	#	Ion Formula	m/z	err [ppm]	mSigma	# mSigma	Score	rdb	e¯ Conf	N-Rule	Adduct
383.1080	1	C19H20NaO7	383.1101	5.5	23.4	1	100.00	10.0	even	ok	M+Na

Figure S49. UV spectrum of compound **5.**

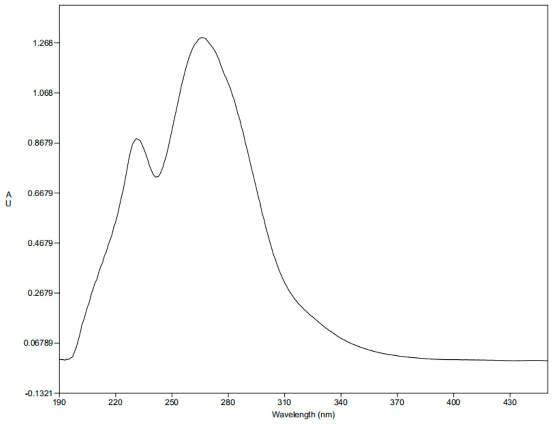

Bio-Kine Software V4.80 Date : 2022/10/23 Time : 1:04:25

COMMENTS :
File name : e:\2022年数据\郭志凯 课题组\zc\zc-14 0.15 mg ml\zc-14_uv.bka
MOS-500 Spectrometer
Spectrum measurement
Acq duration = 0.2 s
Fentes bandwidth = .5 nm
HV = 173 V
Biokine V4.80

Figure S50. IR spectrum of compound **5.**

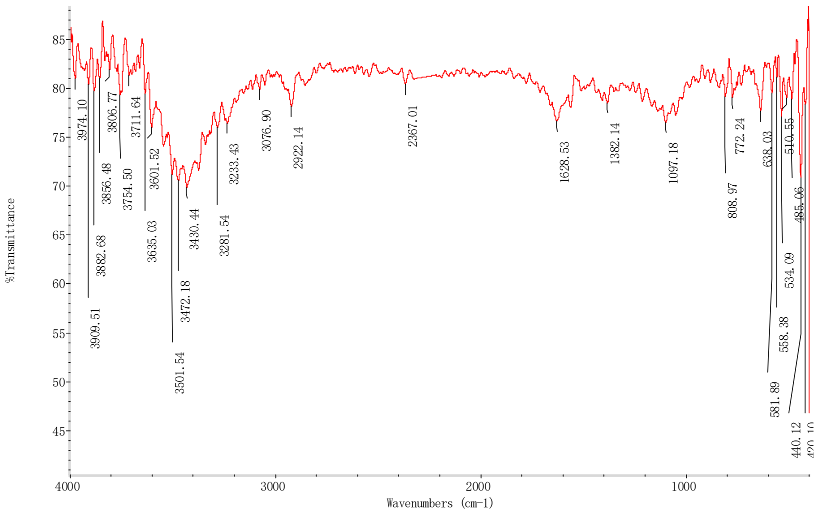

Figure S51. Predicted biosynthetic pathway for compound **5.**

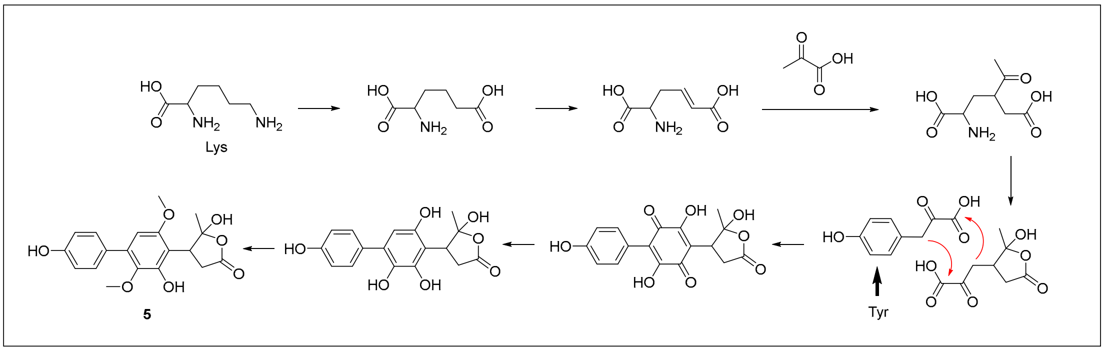

Supplement: Supplementary file 1 [file marinedrugs-20-00679-s001.zip › marinedrugs-1988917-supplementary.pdf]
